# Actin depolymerizing factor ADF7 inhibits actin bundling protein VILLIN1 to regulate root hair formation in response to osmotic stress in *Arabidopsis*

**DOI:** 10.1371/journal.pgen.1010338

**Published:** 2022-09-12

**Authors:** Shuangtian Bi, Mingyang Li, Caiyuan Liu, Xiaoyu Liu, Jianing Cheng, Lu Wang, Jinshu Wang, Yanling Lv, Ming He, Xin Cheng, Yue Gao, Che Wang

**Affiliations:** 1 College of Bioscience and Biotechnology, Shenyang Agricultural University, Shenyang, China; 2 Vegetable Research Institute of Liaoning Academy of Agricultural Sciences, Shenyang, China; Peking University, CHINA

## Abstract

Actin cytoskeleton is essential for root hair formation. However, the underlying molecular mechanisms of actin dynamics in root hair formation in response to abiotic stress are largely undiscovered. Here, genetic analysis showed that actin-depolymerizing protein ADF7 and actin-bundling protein VILLIN1 (VLN1) were positively and negatively involved in root hair formation of *Arabidopsis* respectively. Moreover, RT-qPCR, GUS staining, western blotting, and genetic analysis revealed that ADF7 played an important role in inhibiting the expression and function of VLN1 during root hair formation. Filament actin (F-actin) dynamics observation and actin pharmacological experiments indicated that ADF7-inhibited-VLN1 pathway led to the decline of F-actin bundling and thick bundle formation, as well as the increase of F-actin depolymerization and turnover to promote root hair formation. Furthermore, the F-actin dynamics mediated by ADF7-inhibited-VLN1 pathway was associated with the reactive oxygen species (ROS) accumulation in root hair formation. Finally, ADF7-inhibited-VLN1 pathway was critical for osmotic stress-induced root hair formation. Our work demonstrates that ADF7 inhibits VLN1 to regulate F-actin dynamics in root hair formation in response to osmotic stress, providing the novel evidence on the F-actin dynamics and their molecular mechanisms in root hair formation and in abiotic stress.

## Author summary

Root hairs are required for plants to absorb nutrients and water. The dynamics of cytoskeleton such as actin filaments (F-actin) are necessary for the formation of root hairs, which is regulated by different kinds of cytoskeleton-binding proteins. At the same time, the dynamics of cytoskeleton are also involved in plant abiotic stress tolerance. However, there are few studies on the underlying molecular mechanisms of F-actin dynamics in root hair formation in response to abiotic stress. Actin depolymerization factor 7 (ADF7) and actin bunding protein Villin 1 (VLN1) are important actin-binding proteins in Arabidopsis. Here, we describe a pathway that ADF7 inhibits VLN1 to regulate F-actin dynamics in root hair formation in response to osmotic stress, providing a new evidence for the studies on the molecular mechanisms of F-actin dynamics in root hair formation and in plant abiotic stress tolerance.

## Introduction

Root hair is used as an ideal model to study plant cell elongation and differentiation. Root hairs occupy 77% of the root surface area as the critical water and nutrient absorption site during plant growth, development, and stress management [1]. Under normal conditions, hair cells (H cells), rather than non-hair cells (N cells), in the root epidermal cells rapidly differentiate into root hairs [2–4]. Underwater and nutrient deficiency conditions, plants grow excessively numerous root hairs in response to environmental stresses [5–8].

The *Arabidopsis* actin single mutant *act2* and *act7* seedlings show fewer root hairs, and double mutant *act2 act7* seedlings display full defects in root hair formation, indicating that actin cytoskeleton is required for root hair formation [3,9–11]). Before root hair formation, F-actin dynamics show that numerous longitudinal filament actin (F-actin) (parallel growth axes) surrounds the nuclei near the end walls in root epidermal cells of the root hair emission region in the wild type (WT) [12,13]. In the root epidermal cells of *act2 act7*, the thicker and more transversely oriented F-actin bundles or rod-like structures instead of the finer and more longitudinal F-actin in that of WT seedlings [10]. This indicates that the changes of F-actin architecture including the F-actin orientation and thickness in root epidermal cells are closely related to root hair formation.

A few actin-binding proteins (ABPs) are identified in root hair initiation. Profilin plays a primary role in accelerating F-actin assembly; additionally, it promotes actin bundles/cables and inhibits actin nucleation [14–19]. *Arabidopsis profilin1* mutant, *prf1-1*, seedlings develop higher density and longer root hairs [15], suggesting that PRF1 is involved in root hair formation and elongation. CROOKED/ARPC5 is molecularly identified as a subunit of the ARP2/3 complex that possesses the function of nucleating actin assembly [20,21]. *Crooked* seedlings grow more than one root hair from the same hair cell, suggesting that ARPC5 negatively regulates root hair formation [21]. AtFH8 participates in several cytoskeletal functions such as nucleating, capping, binding, severing F-actin, and binding to profilin [22,23]. Overexpression of *AtFH8* leads to producing more than one root hair on one hair-forming site, indicating that FH8 is involved in bulges formation rather than root hair formation [23]. Because the actin arrays and dynamics mediated by PRF1 and ARPC5 in root hair formation are not reported, how PRF1 and ARPC5 regulate actin dynamics to affect root hair formation remained unknown. Therefore, the molecular mechanisms of actin dynamics mediated by ABPs in root hair formation are largely unknown.

Actin depolymerization factors (ADFs) are responsible for de-polymerizing and severing single F-actin [24–27]. *Arabidopsis* ADF7 is one of 11 ADF family proteins and possesses the mild activities of single F-actin depolymerizing and severing, compared with the other ADFs [24,28]. Previous findings showed that ADF7 is required for pollen tip growth by severing actin-mediated turnover of F-actin [28]. Additionally, ADF7 highly expresses in the microspore stage using *ADF7-*GFP expression analysis [25]. Villins (VLNs) prominently possess F-actin bundling abilities [29–31]. *Arabidopsis* genome encodes 5 VLN isoforms (VLN1-5) [29,32]. VLN1 displays a simple actin bundling capacity in a calcium ion (Ca^2+^) and Ca M in an independent manner [32]. VLN1 is highly expressed in various plant tissues including leaves, hypocotyls, roots, and root hairs [29,33]. VLN1 and VLN3 play a partially overlapping role in the turnover of actin bundle formation *in vitro* [34]. VLN1 interacts with ADFs to affect F-actin dynamics *in vitro* [32]. Additionally, VLN1 negatively regulates root hair elongation mediated by transcription factor GL2 in osmotic stress [33].

ROS accumulation plays an important role in root hair formation and elongation [35–38]. In plants, NADPH oxidase catalyzes ROS production. NADPH oxidase-mediated ROS production is the best-characterized mechanism during root hair development [35–37]. NADPH oxidase is encoded by ROOT HAIR DEFECTIVE 2 (RHD2/RBOHC) gene [35,36]. *rhd2* mutants display defects in root hair formation and elongation, correlated with reduced ROS levels in roots and root hairs [35–37]. FER, FERONIA receptor-like kinase, is required for root hair development by regulating NADPH oxidase-dependent ROS production in root and root hairs [37]. Actin dynamics are involved in the regulation of ROS level *in vivo* [26,39]. Both moderate actin polymerization and moderate actin depolymerization increase NADPH oxidase activity in microglia [40]. F-actin depolymerization elevates ROS levels in roots by regulating *RHD2* expression in salt stress in *Arabidopsis* [39]. Actin disrupting drug Lat B resulted in a significant increase in immunogenic peptide *flg22*-induced ROS production [26]. Therefore, we suppose that the integration of the actin dynamics and ROS signaling might play a key role during root hair development. Here, we found that ADF7 inhibited the expression and the function of VLN1, resulting in elevating F-actin depolymerization, fine F-actin amount, F-actin turnover, and ROS accumulation in root epidermal cells and new emerged root hair cells, which plays an important role in osmotic stress-induced root hair formation, providing the first evidence on the molecular mechanisms of F-actin depolymerization and F-actin bundling in root hair formation and in osmotic stress.

## Results

### Actin-depolymerizing protein ADF7 is positively involved in root hair formation

To explore the role of ADF7 in root hair formation, we first identified T-DNA insert mutants *adf7-2* and constructed complementation lines (*ADF7*-comps) by transforming *ADF7* promoter (p*ADF7*)::*ADF7* in *adf7-2* plants and overexpressing lines (*ADF7* OEs) by transforming *35S*::*ADF7* in Col-0 plants (S1 Fig)). Reverse transcription-PCR (RT-PCR) and RT-quantitative PCR (RT-qPCR) analyses showed no detectable expression in *adf7-2*, indicating that *adf7-2* is a knock-out line, and Col-like expression in *ADF7* comp #2 and #7, ~2.3-fold higher expression in *ADF7* OE #13, and~3.1-fold higher in *ADF7* OE #14 (S1B–S1E Fig).

**Fig 1:**
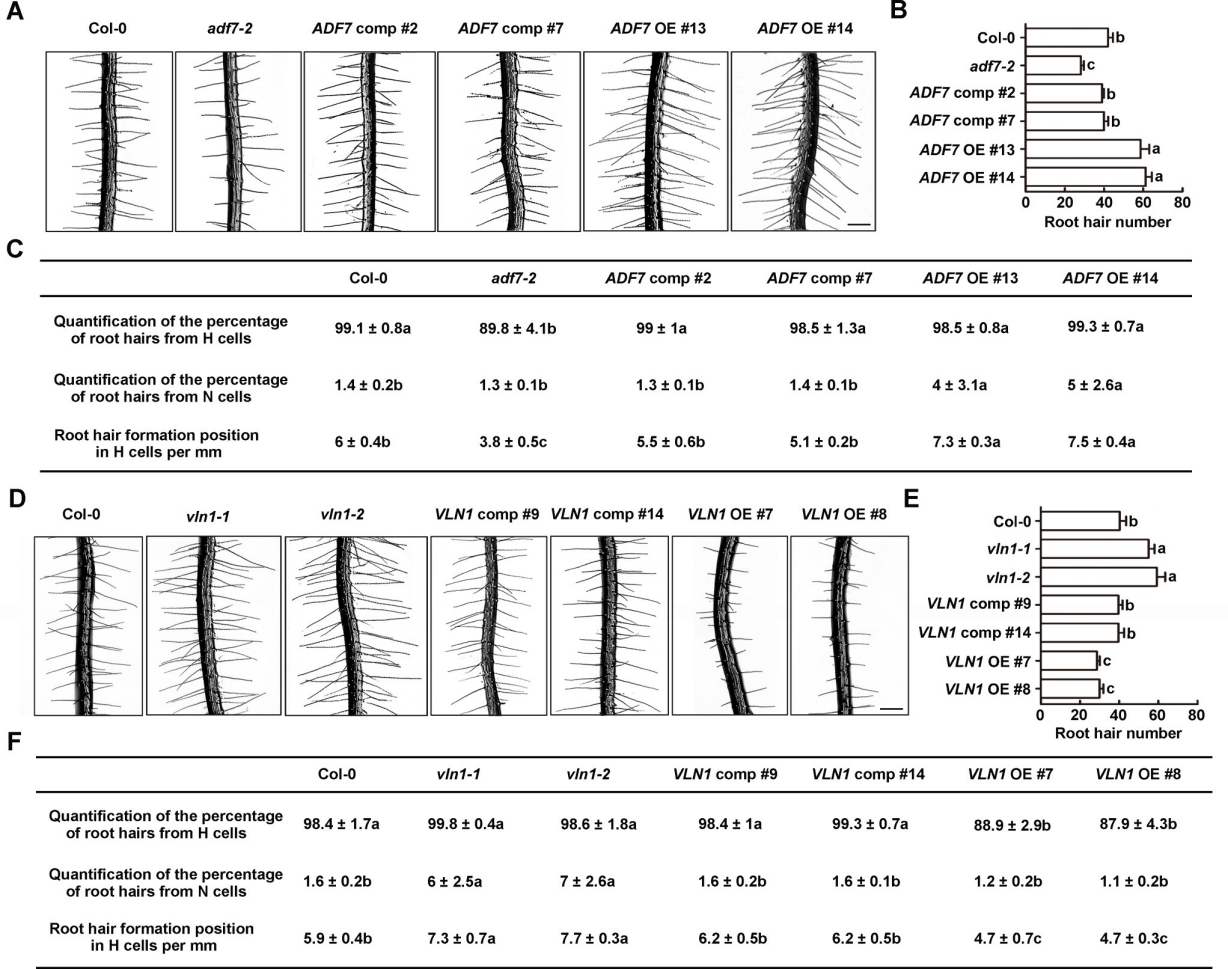
Actin depolymerization protein ADF7 is positively involved in root hair formation and actin bunding protein VLN1 is negatively involved in root hair formation. **(A)** Images of root hairs from wild type (Col-0), *adf7-2*, *ADF7* comp #2, *ADF7* comp #7, *ADF7* OE #13, and *ADF7* OE #14. Scale bar, 200 μm. OE, overexpression. **(B)** Histogram depicting root hair number in **(A)**. **(C)** Quantification of the percentage of root hairs from H and N cells and root hair formation position in H cells in **(A)**. Values given are means ± SD. **(D)** Images of root hairs from Col-0, *vln1-1*, *vln1-2*, *VLN1* comp #9, *VLN1* comp #14, *VLN1* OE #7, and *VLN1* OE #8. Scale bar, 200 μm. **(E)** Histogram depicting root hair number in **(D)**. **(F)** Quantification of the percentage of root hairs from H and N cells and root hair formation position in H cells in **(D)**. Values given are means ± SD. Significant difference (*P*< 0.05) indicated by different letters among genotypes is determined for each condition by one-way ANOVA followed by Tukey’s test in **(B)**, **(C)**, **(E)**, and **(F)**.

Then, we calculated root hair numbers in Col-0, *adf7-2*, *ADF7* comp #2, *ADF7* comp #7, *ADF7* OE #13, and *ADF7* OE #14, as described previously [4]. Our results showed that Col-0 seedlings grew ~40 root hairs, similar to the previous report [4]. *adf7-2* displayed~28 root hairs, respectively. The defects in root hair number in *adf7* was rescued in *ADF7* comp #2 and #7 (Fig 1A and 1B), confirming that *ADF7*’s loss-of-function caused the root hair number phenotype in *adf7*. In contrast, both *ADF7* OE #13 and #14 seedlings had ~60 root hairs (Fig 1A and 1B).

Next, we calculated the percentage of root hairs from H and N cells in *ADF7* genotype seedlings using the previous method [41]. Col-0 seedlings grew ~99% hairs from H cells and ~1% hair from N cells. Compared with Col-0, *adf7-2* showed decreased root hair numbers from H cells and no significant change from N cells, while *ADF7* OE #13 and #14 seedlings showed no change in root hair number from H and the increased number from N cells (Fig 1C). These results indicated that ADF7 is positively involved in the differentiation of H and N cells in root hair initiation. To explore root hair formation position from H cells, we calculated the root hair number along a line of H cells in a fixed zone between 2.5 and 3.5 mm from the primary root tips in Col-0 and *ADF7* genotype seedlings according to the previous method [42]. The results mean that the density of H cells in the root epidermal cells from various genotype seedlings. Our results showed that Col-0 seedlings grew ~6 root hairs per mm along with H cells (Figs 1C and S2 similar to the previous report [42]. Compared with Col-0, *adf7* mutants showed a decreased root hair number along with H cells, whereas *ADF7* OE seedlings displayed an increased number (Figs 1C and S2). The results showed that ADF7 is involved in increasing the density of H cells in the root epidermal cells. The results illustrate that ADF7 plays a positive role in root hair formation.

### Actin-bundling protein VLN1 is negatively involved in root hair formation

To explore whether VLN1 is involved in root hair formation, *vln1-1*, *vln1-2*, *VLN1* comp #9 and #14 and *VLN1* OE #7 and #8 were used ([33], S3 Fig) to calculated three parameters including root hair number in roots, the percentage of root hairs from H and N cells, and root hair number along H cells, using the same methods mentioned above. The results showed that *vln1* mutants (~ 60 root hairs) grew more root hair numbers than Col-0 (~ 40 root hairs) (Fig 1D and 1E), associated with *vln1* displayed a higher percentage of root hairs from N cells and more root hairs along H cells (Figs 1F and S2). These root hair phenotypes of *vln1* mutants were restored in *VLN1* comp #9 and #14 (Fig 1D–1F). *VLN1* OE #7 and #8 seedlings displayed ~ 30 root hairs, associated with a lower percentage of root hairs from H cells and fewer root hairs along H cells (Figs 1 and S2). These results indicate that VLN1 is negatively involved in root hair initiation.

### ADF7 inhibits the expression and function of VLN1 during root hair formation

**Fig 2:**
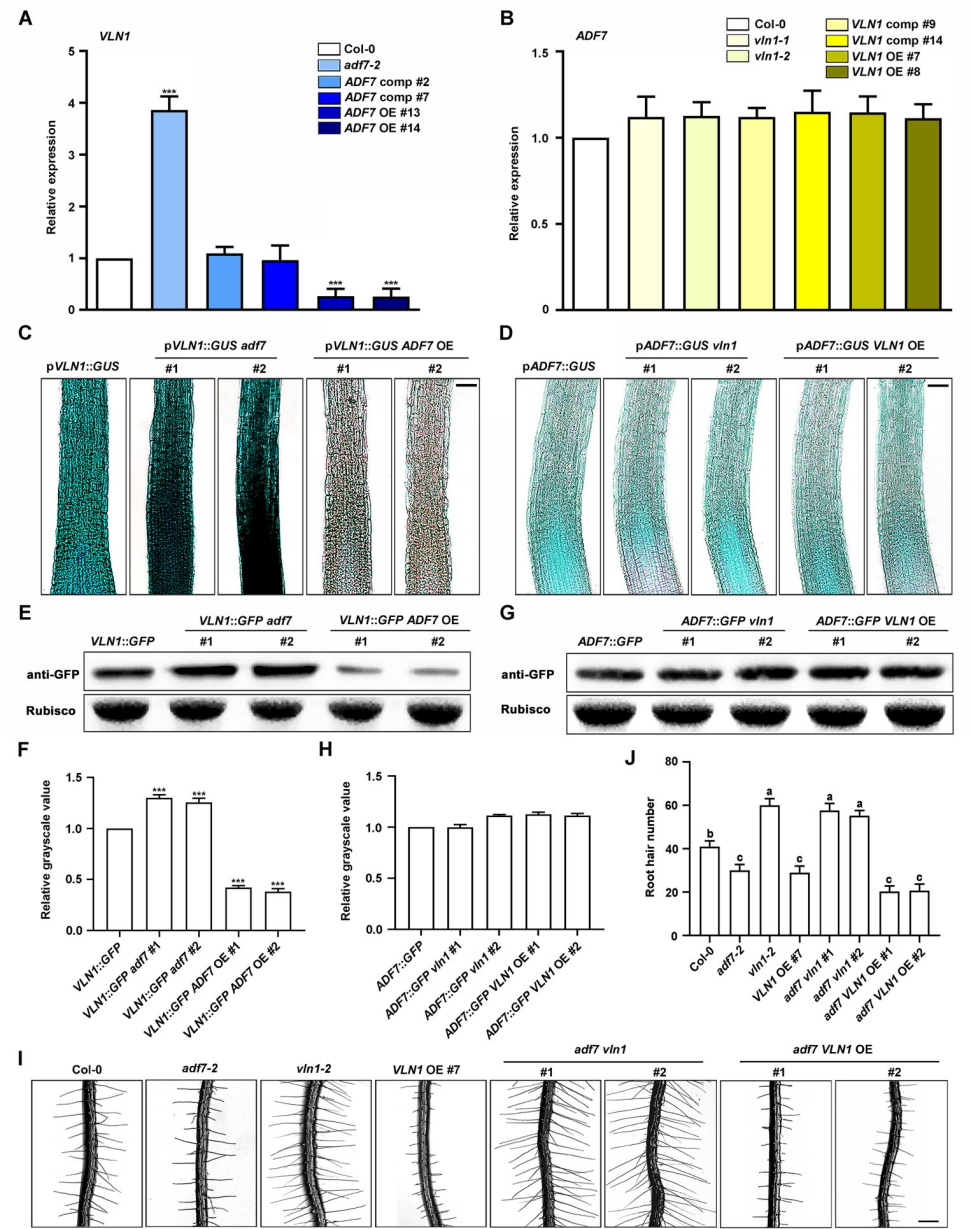
ADF7 inhibits the expression of function VLN1 in root hair formation. **(A)** RT-qPCR quantification of *VLN1* expression level in Col-0, *adf7-2*, *ADF7* comp #2, *ADF7* comp #7, *ADF7* OE #13, and *ADF7* OE #14. **(B)** RT-qPCR quantification of *ADF7* expression level in Col-0, *vln1-1*, *vln1-2*, *VLN1* comp #9, *VLN1* comp #14, *VLN1* OE #7, and *VLN1* OE #8. **(C)** *GUS* analysis of *VLN1* expression in root tips from Col-0, *adf7-2*, and *ADF7* OE seedlings. **(D)** *GUS* analysis of *ADF7* expression in root tips from Col-0, *vln1*, and *VLN1* OE seedlings. **(E)** Western blotting of VLN1 expression level in Col-0, *adf7-2*, and *ADF7* OE seedlings. Rubisco as a loading control. **(F)** Quantification of the relative grayscale value in (E). **(G)** Western blotting of ADF7 expression level in Col-0, *vln1*, and *VLN1* OE seedlings. Rubisco as a loading control. **(H)** Quantification of the relative grayscale value in (G). **(I)** Images of root hairs from *ADF7* and *VLN1* double gene genotypes. Scale bar, 200 μm. **(J)** Histogram depicting root hair number in **(I)**. Significant difference (*P*< 0.05) indicated by different letters among different genotypes is determined for each condition by one-way ANOVA followed by Tukey’s test. Values are means ± SD of three independent biological replicates. *** *P*< 0.001, Student’s *t*-test compared to Col-0, in **(A)** and **(B)**, and compared to *VLN1*::GFP and *ADF7*::GFP in **(F)** and **(H)**.

Next, we investigate whether ADF7 and VLN1 interact during root hair formation. RT-qPCR analysis showed that *VLN1* expression in roots was significantly increased in *adf7* mutants, but inhibited in *ADF7* OE seedlings (Fig 2A). While *ADF7* expression in roots wasn’t significantly changed in *vln1* mutants and *VLN1* OEs (Fig 2B). Additionally, we generated the GUS staining genotype seedlings by introducing the *VLN1* promoter (p*VLN1*)::*GUS* into *adf7-2* and *ADF7* OE #13, respectively, and introducing the *ADF7* promoter (p*ADF7*)::*GUS* into *vln1-2* and *VLN* OE #8 by crossing, respectively. We also introduced a p*VLN1*::*VLN1*::*GFP* and a p*ADF7*::*ADF7*::*GFP* into *vln1-2* and *adf7-2*, respectively, which results in fully rescuing the root hair phenotypes of *vln1-2* and *adf7-2*, respectively (S4 and S5 Figs), indicating that the GFP fusion protein may be used in protein expression analysis. Analysis of GUS staining also showed that VLN1 doesn’t affect ADF7 expression in roots and ADF7 negatively regulates VLN1 expression in roots (Fig 2C–2H). Further, western blotting supported that ADF7 affects VLN1 expression in roots (Fig 2C–2H). Further, we generated *ADF7* and *VLN1* double gene genotype seedlings, including *adf7 vln1* #1 and #2, and *adf7 VLN1* OE #1 and #2 from crossing *adf7-2*, *vln1-2*, and *VLN1* OE #8 respectively (S6 Fig). Compared with Col-0, *adf7 vln1* seedlings displayed more root hair number, similar to single *vln1* mutants (Fig 2I and 2J). In *adf7 VLN1* OE seedlings had contrasting results, similar to *VLN1* OE seedlings (Fig 2I and 2J). The results confirm that ADF7 is upstream of VLN1 in root hair formation, and ADF7 inhibits the expression and function of VLN1 in root hair formation.

### ADF7 inhibits VLN1-mediated thick bundle formation

**Fig 3:**
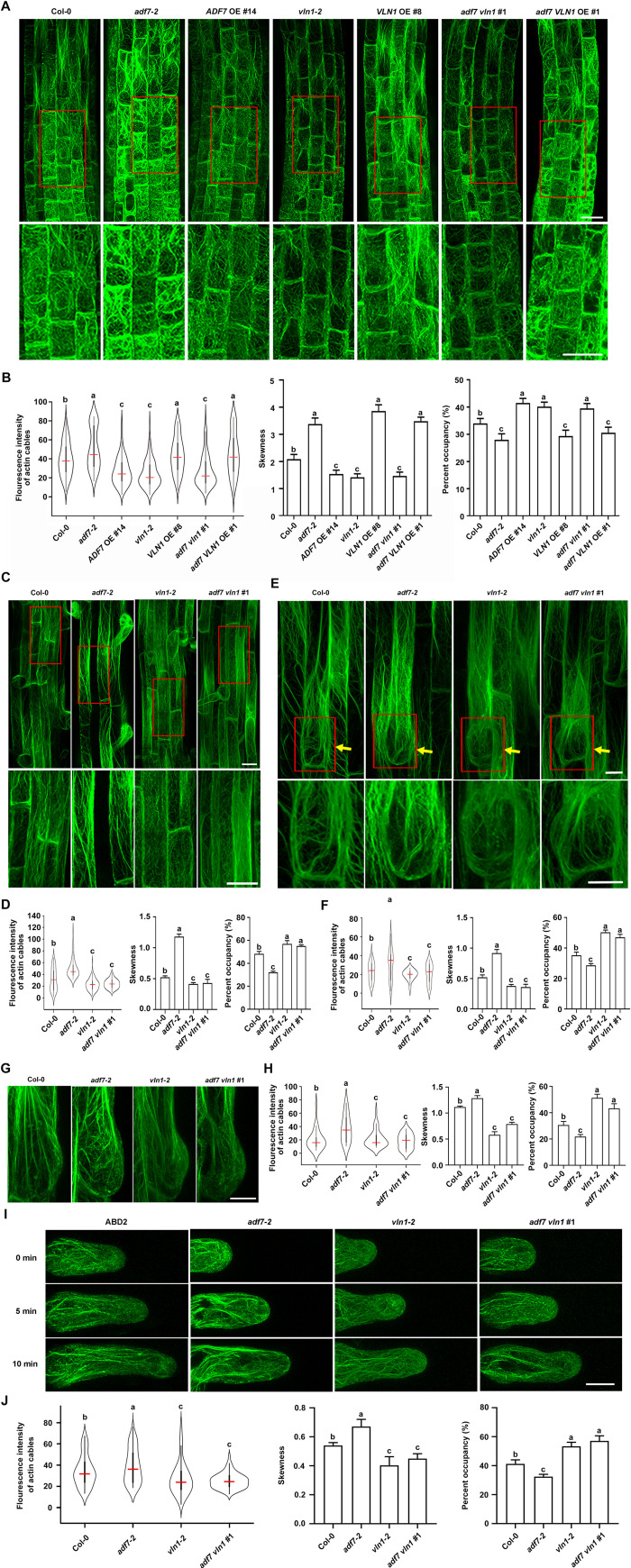
ADF7 inhibits VLN1-mediated thick bundle formation in the root epidermal cells from different regions and outgrowing root hairs. **(A)** Confocal microscopy images of root epidermal cells visualized by the expression of *fABD2*::*GFP* in the elongation/differentiation and transition regions from Col-0, *adf7-2*, *ADF7* OE #14, *vln1-2*, *VLN1* OE #8, *adf7 vln1* #1, and *adf7 VLN1* OE #1 seedlings. Enlarged views from the red boxes are in the bottom row. Scale bar, 20 μm. **(B)** Violin plot showing the average and contribution of fluorescence intensity of actin cables in **(A)**. The red line represents the average fluorescence intensity in different genotypes. Histogram depicting skewness and percentage of occupancy of F-actin in **(A)**. **(C)** Confocal microscopy images of root epidermal cells in the root hair region from the primary root tips of 4-d-old seedlings in **(A)**. Enlarged views from the red boxes are in the bottom row. Scale bar, 25 μm. **(D)** The parameter fluorescence intensity of actin cables, skewness, and percentage of occupancy of F-actin in **(C)**. **(E)** Confocal microscopy images of outgrowing root hair cells from the primary root tips of 4-d-old seedlings in Col-0, *adf7-2*, *vln1-2*, and *adf7 vln1* #1 in cross-section. Enlarged views from the red boxes are in the bottom row. Scale bar, 20 μm. **(F)** The parameter fluorescence intensity of actin cables, skewness, and percentage of occupancy of F-actin in **(E)**. **(G)** Confocal microscopy images of outgrowing root hair cells from the primary root tips of 4-d-old seedlings in Col-0, *adf7-2*, *vln1-2*, and *adf7 vln1* #1 in the longitudinal section. Scale bar, 10 μm. **(H)** The parameter fluorescence intensity of actin cables, skewness, and percentage of occupancy of F-actin in **(G)**. **(I)** Time-lapse images of newly emerged root hairs of Col-0, *adf7-2*, *vln1-2*, and *adf7 vln*1 #1 for 0, 5, and 10 min. Scale bar, 10 μm. **(J)** The parameter fluorescence intensity of actin cables, skewness, and percentage of occupancy of F-actin in **(I)**. At least 60 root epidermal cells from at least 20 individual seedlings are calculated in every genotype. Significant difference (*P*< 0.05) indicated by different letters among genotypes is determined for each condition by one-way ANOVA, followed by Tukey’s test in **(B)**, **(D)**, **(F)**, **(H)**, and **(J)**.

**Fig 4:**
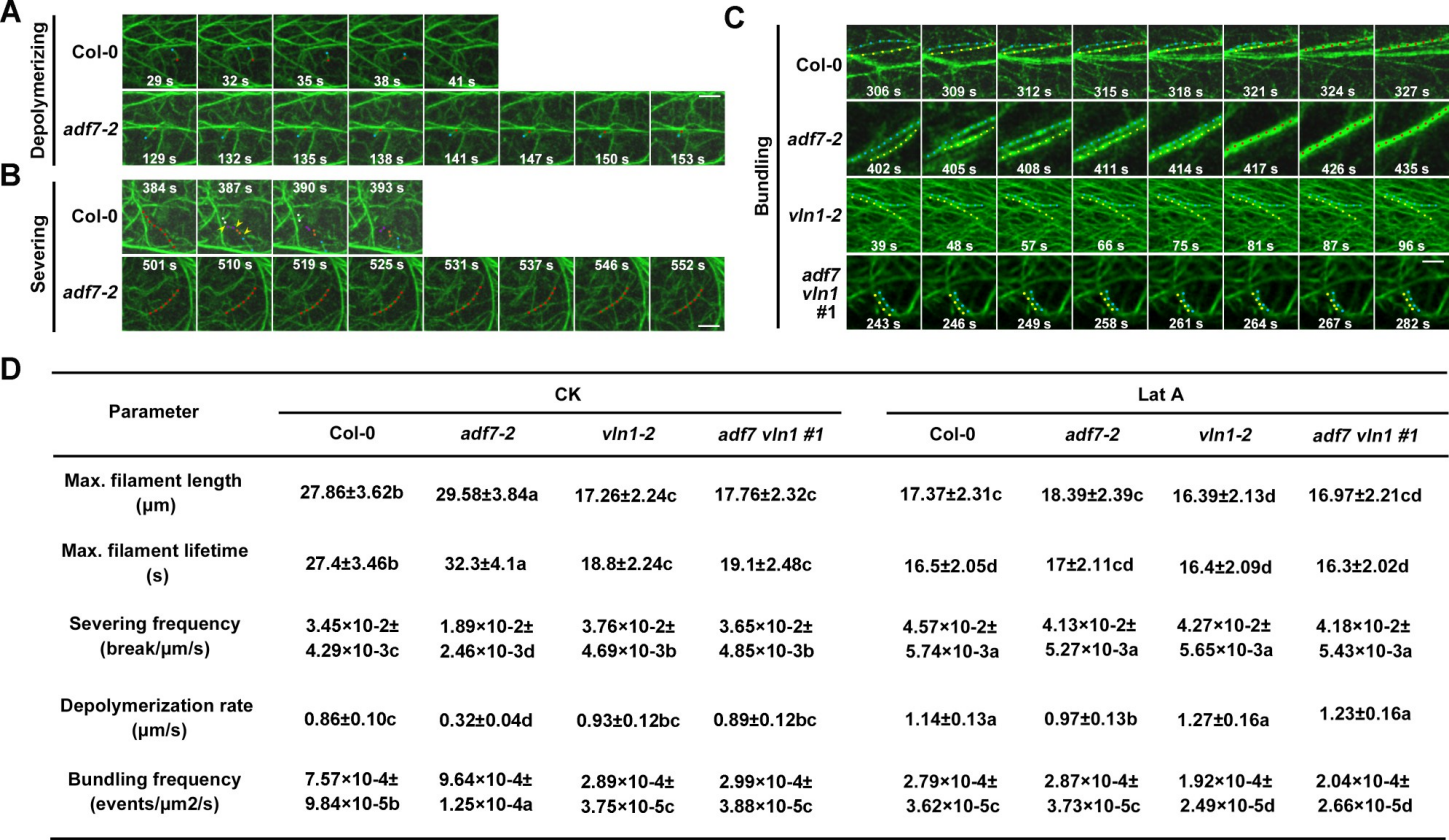
The F-actin dynamics regulated by ADF7-inhibited-VLN1 on single filament level in epidermal cells of root apices. **(A)** Depolymerizing processes in Col-0 and *adf7-2* seedlings. Scale bar, 10 μm. **(B)** Severing processes in Col-0 and *adf7-2* seedlings. Scale bar, 10 μm. **(C)** Bundling processes in Col-0, *adf7-2*, *vln1-2*, and *adf7 vln1*#1 seedlings. Scale bar, 10 μm. **(D)** The parameters of actin dynamics regulated by ADF7 and VLN1 on single actin filament level in epidermal cells of root spices under normal conditions and Lat A treatments. To analyze the bundling frequency, a 30×30 μm^2^ region was selected, At least 60 root cells from at least 20 individual seedlings are calculated in every genotype. Significant difference (*P*< 0.05) indicated by different letters among genotypes is determined for each condition by one-way ANOVA followed by Tukey’s test.

To explore F-actin arrays and dynamics mediated by ADF7 and VLN1 during root hair formation, we observed F-actin arrays and dynamics of *ADF7* and *VLN1* genotype seedlings (Figs 3A and 4). Because root hairs grow out of the root epidermal cells of the elongation/differentiation region, the observed regions were first chosen in the elongation/differentiation and transition regions from the primary root tips of 6-d-old seedlings, according to the abovementioned method [43]. F-actin is visualized by expressing an ideal actin filamentous-specific fluorescent probe, *fABD2*::*GFP* [44,45]. To quantify the actin organization, the skewness parameter indicates the extent of thick bundles and the percentage of occupancy to estimate the density of actin bundles (amount of F-actin) [45]. Additionally, we used a violin plot to analyze the average and contribution of the GFP fluorescence intensity of the actin cables to estimate F-actin thickness further (Fig 3A and 3B). High fluorescence intensity peaks represent brightly labeled actin bundles (generally from thick actin bundles), whereas low peaks represent weakly labeled actin filament bundles (generally from fine actin bundles) [46].

Col-0 exhibited preferentially parallel growth axes longitudinal and oblique F-actin in the elongation/differentiation region but lost the preferential F-actin arrays, displaying longitudinal, oblique, and lateral short F-actin bundles in the transition region where cells showed square and close to square (Fig 3A and 3B). Compared with Col-0, the different observed genotypes showed similar F-actin orientation but significant differences in thickness and density of F-actin. *adf7* and *VLN1* OE showed increased thick bundles and declined amount of F-actin, consistent with higher fluorescence intensity, higher skewness parameter, and the lower percentage of occupancy (Fig 3A and 3B). In contrast, *vln1* and *ADF7* OE seedlings displayed the decreased thick bundles and the increased F-actin density, compared with Col-0 (Fig 3A and 3B). Additionally, *vln1* and *ADF7* OE seedlings had numerous actin filaments longer than 15 μM (Figs 3A and 4D), suggesting that VLN1 loss-of-function or ~3-fold increased *ADF7* expression *in vivo* lead to mild actin depolymerization, rather than severe disruption of F-actin. Moreover, *adf7 vln1* showed a similar F-actin architecture to *vln1*, and *adf7 VLN1* OE and *VLN1* OE seedlings were similar (Fig 3A and 3B).

Additionally, we observed F-actin architecture in root epidermal cells of the root hair region from the primary root tips of 4-d-old seedlings, outgrowing bugles from the primary root tips of 4-d-old seedlings in cross or longitudinal sections, and new emerged root hair cells from the primary root tips of 4-d-old seedlings (Fig 3C–3J). Col-0 exhibited preferentially longitudinal F-actin in the root hair region. *adf7*, *vln1*, and *adf7 vln1* seedlings also showed a visible longitudinal F-actin but a significant difference in the thickness and density of F-actin with Col-0 (Fig 3C and 3D). Compared with Col-0, *adf7* showed thicker bundles and less F-actin, whereas *vln1* and *adf7 vln1* displayed contrasting results (Fig 3C and 3D). In root epidermal cells with outgrowing root hairs, Col-0 exhibited preferentially longitudinal F-actin assembly nearly ahead of the root hair formation site (shown with yellow arrows) in the cross-section (Fig 3E and 3F). In the longitudinal section of Col-0 outgrowing root hairs, several longitudinal F-actin bundles of root epidermal cells extended to the outgrowing bugles, and short and fine F-actin diffused visible fluorescence (short and fine F-actin) in the bugles (Fig 3E and 3F). F-actin dynamics in the new emerged root hair cells in Col-0 display a change from confused orientation to several longitudinal F-actin bundles (parallel root hair growth axes) during the root hair cell elongation. *adf7*, *vln1*, and *adf7 vln1* seedlings showed a difference in the thickness and density of F-actin with Col-0 was in the outgrowing bugles and the new emerged root hair cells (Fig 3G–3J). These results illustrate that ADF7 inhibits VLN1-mediated thick bundle formations, leading to the changes in the thickness and density of F-actin, in root epidermal cells, outgrowing bugles and new emerged root hair cells.

### ADF7 inhibits VLN1-mediated thick bundling activity in epidermal cells of root apices

F-actin arrays are correlated with the organization of single actin filament dynamics [47]. Previous findings have shown that ADF7 possesses actin severing and depolymerizing capacities *in vitro* and in pollen cells, and VLN1 displays a simple mechanism of bundling capacity *in vitro* and in root hairs [28,32–34]. Therefore, we put our efforts toward characterizing the actin organization capacities of ADF7 and VLN1 in root apices. The observed regions were the elongation zone of root apices (Fig 4). The results showed that ADF7 loss-of-function led to a decline in F-actin severing frequency and depolymerization rate and an increase in bundling frequency, as well as the decreased F-actin turnover based on the increased maximum filament length and maximum filament lifetime, and the decreased severing frequency and depolymerization rate (Fig 4 and S1–S6 Videos). *VLN1* mutation showed contrasting results (Fig 4 and S7 Video). Moreover, *adf7 vln1* double mutants displayed similar F-actin dynamics to *vln1* (Fig 4 and S8 Video). These results illustrate that ADF7 is responsible for organizing single F-actin depolymerizing and severing, and VLN1 functions in single F-actin bundling. The results also indicate that ADF7 inhibits VLN1-mediated single F-actin bundling, which leads to a significant increase of F-actin turnover, in root apices.

### ADF7-inhibited-VLN1 pathway activates root hair formation by regulating F-actin dynamics in root tips

**Fig 5:**
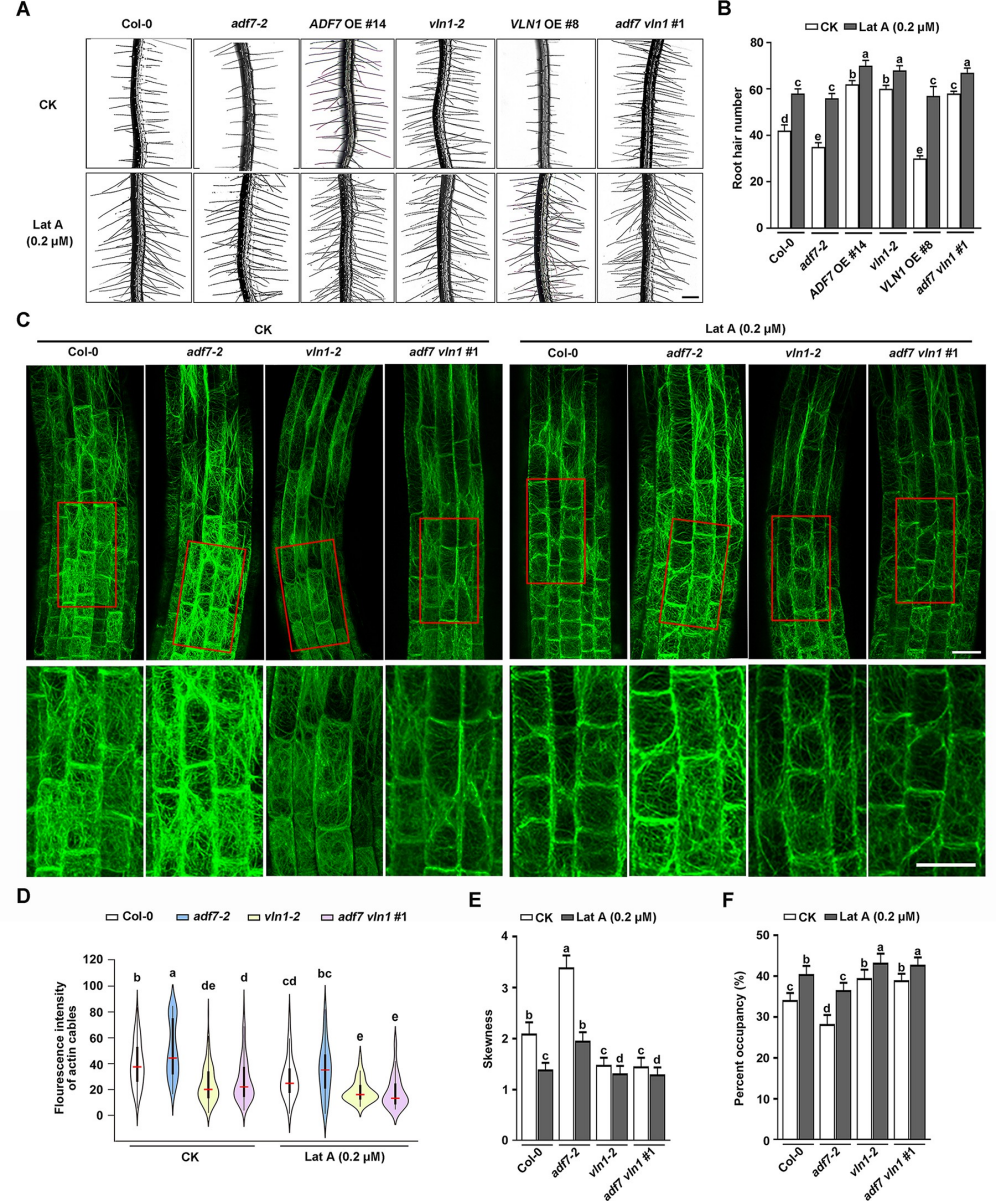
The F-actin depolymerization regulated by ADF7-inhibited-VLN1 promotes root hair formation. **(A)** Images of root hairs from Col-0, *adf7-2*, *ADF7* OE #14, *vln1-2*, *VLN1* OE #8, and *adf7 vln1* #1 under CK and Lat A treatments (0.2 μM). Scale bar, 200 μm. **(B)** Histogram depicting root hair number in **(A)**. **(C)** Confocal microscopy images of epidermal cells visualized by the expression of *fABD2*::*GFP* in root apices from Col-0, *adf7-2*, *vln1-2*, and *adf7 vln1* #1 seedlings under Lat A treatments (0.2 μM). Enlarged views from the red boxes are in the bottom row. Scale bar, 25 μm. **(D)** Violin plot showing the average and contribution of fluorescence intensity of actin cables in **(C)**. The red line represents the average fluorescence intensity in different genotypes. **(E)** Histogram depicting skewness of F-actin in **(C)**. **(F)** Histogram depicting the percentage of occupancy of F-actin in **(C)**. Significant difference (*P*< 0.05) indicated by different letters among genotypes is determined for each condition by one-way ANOVA followed by Tukey’s test in **(B)**, **(D)**, **(E),** and **(F)**.

Considering ADF7 and VLN1 can bind to actin *in vitro* [28,32], and the direct roles of ADF7 and VLN1 in controlling actin dynamics [28,32,48,49], therefore, we proposed that ADF7 and VLN1 might be via directly controlling F-actin dynamics to affect root hair formation. Then, we conducted actin pharmacological experiments. We firstly calculated root hair number in Col-0, *adf7-2*, *ADF7* OE #14, *vln1-2*, *VLN1* OE #8, *adf7 vln1* #1, and *adf7 VLN1* OE #1 seedlings from 3-d-old seedlings treated with the presence or absence of actin disrupting drug latrunculin-A (Lat A) for 3 h then removed in no drug media for 3-d growth. In Col-0 seedlings, the low concentrations of Lat A (0.2 and 0.4 μM) stimulated significant root hair formation (Fig 5A and 5B and S7). Moreover, Lat A treatments rescued the defects in root hair formation in *adf7*, *VLN1* OE, and *adf7 VLN1* OE seedlings (Figs 5A and 5B and S7), illustrating that actin depolymerization activates ADF7-inhibited-VLN1-regulated root hair formation. No significant difference between 0.2 and 0.4 μM Lat A treatments was found (Figs 5A and 5B and S7).

Next, we observed the actin dynamics in Col-0, *adf7*, *vln1*, and *adf7 vln1* seedlings under Lat A treatments in root epidermal cells in root tips (Fig 5C). 0.2 μM Lat A treatments led to the decline of fluorescence intensity and skewness parameter and the increase of occupancy percentage, suggesting that actin disrupting drug with low concentrate increased actin depolymerization and inhibited actin thick bundles in Col-0, *adf7*, *vln1*, and *adf7 vln1* seedlings (Fig 5C–5F). Furthermore, the mild actin depolymerization treatments increased F-actin turnover in roots from Col-0, *adf7*, *vln1*, and *adf7 vln1* seedlings (Figs 5C–5F and 4D). These results indicate that actin disrupting drug increased F-actin depolymerization, fine F-actin and F-actin turnover in roots, consequently promoting root hair formation in all the seedlings including Col-0, *ADF7*, and *VLN1* genotype seedlings, highlighting that ADF7-inhibited-VLN1 pathway organizes F-actin dynamics including the increase of F-actin depolymerization, fine F-actin amount and F-actin turnover in root tips to promote root hair formation.

### ADF7-inhibited-VLN1 pathway is associated with the reactive oxygen species (ROS) accumulation in root tips

**Fig 6:**
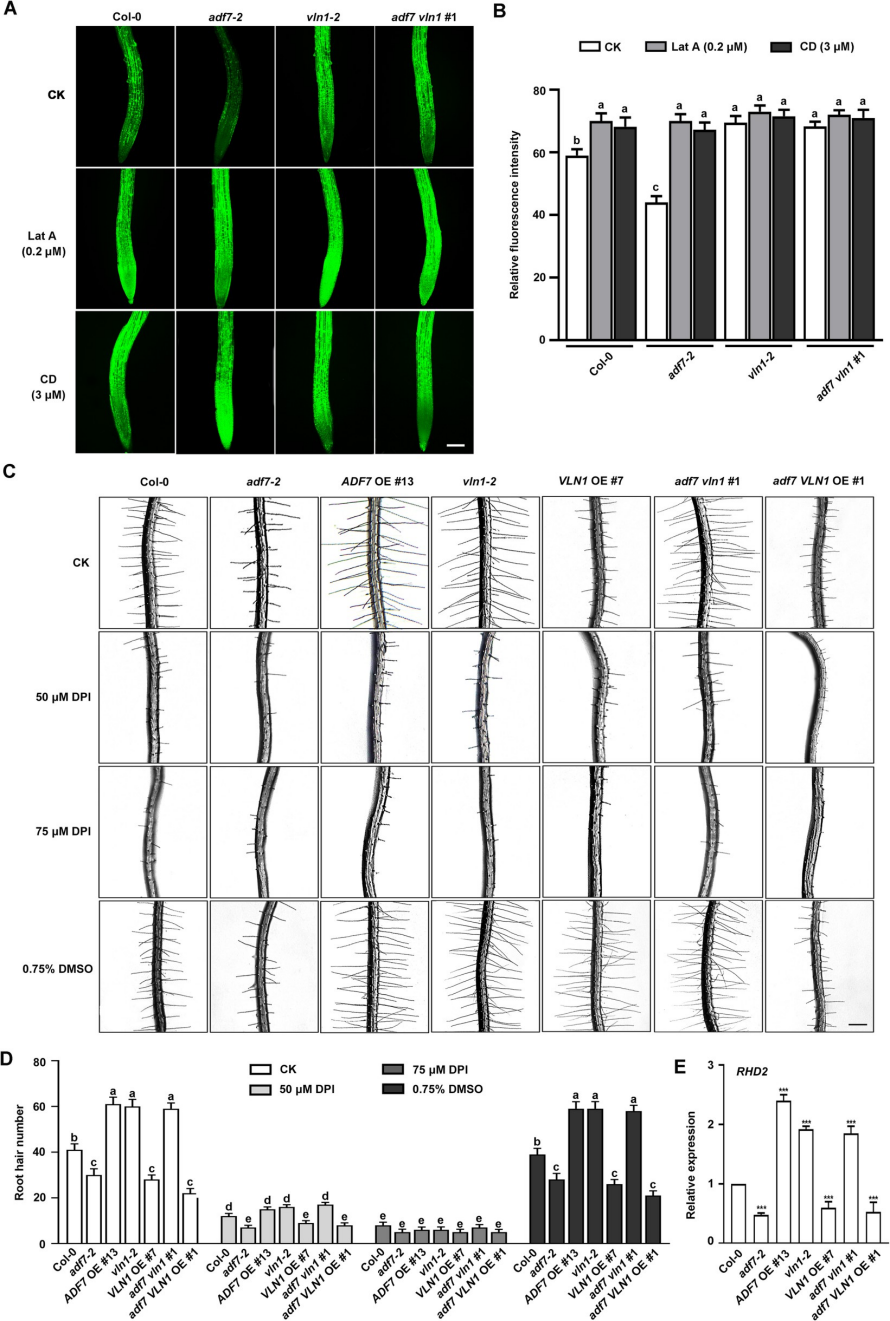
The F-actin depolymerization co-regulated by ADF7-inhibited-VLN1 elevates ROS accumulation during root hair formation. **(A)** Images of ROS levels in roots of Col-0, *adf7-2*, *vln1-2*, and *adf7 vln1* #1 seedlings under Lat A treatments (0.2 μM) and CD (3 μM). Seedlings were treated with H2DCF–DA (see **Methods**), scale bar, 100 μm. **(B)** Histogram depicting relative fluorescence intensities of ROS accumulation in **(A)** At least 30 seedlings were examined for each material in different treatments. **(C)** Images of root hairs from Col-0, *adf7-2*, *vln1-2*, *ADF7* OE #13, *vln1-2*, *VLN1* OE #7, *adf7 vln1* #1, and *adf7 VLN1* OE #1 under 50 μM and 75 μM DPI treatments. A negative control was supplemented with 0.75% DMSO. Scale bar, 200 μm. **(D)** Histogram depicting root hair number in **(C)**. **(E)** RT-qPCR quantification of RHD2 expression level in Col-0, *adf7-2*, *vln1-2*, and *adf7 vln1 #1*. Values are means ± SD of three independent biological replicates. *** *P* < 0.001, Student’s *t*-test compared to Col-0. Significant difference (*P*< 0.05) indicated by different letters among genotypes is determined for each condition by one-way ANOVA, followed by Tukey’s test in **(B)** and **(D)**.

ROS accumulation promotes root hair development [35,37]. F-actin depolymerization is involved in increasing ROS levels *in vivo* [39]. We explored whether the role of ADF7 and VLN1 in root hair formation is associated with ROS accumulation. Then, we first calculated ROS production of 6 d-old roots in Col-0 and *ADF7* and *VLN1* genotype seedlings. Compared with Col-0, ROS level decreased in *adf7*, *VLN1* OE, *adf7* VLN1 OE seedlings, whereas it increased in *ADF7* OE, *vln1*, and *adf7 vln1* seedlings (Fig 6A and 6B), indicating that ADF7-inhibited-VLN1 promoted ROS level in root tips. Next, we conducted actin pharmacological experiments by calculating ROS production in different genotypes under Lat A and Cytochalasin D (CD) treatments. The results showed that 0.2 μM Lat A and 3 μM CD treatments led to the increased ROS levels in Col-0, *adf7*, *vln1*, and *adf7 vln1* roots, further indicating that ADF7-inhibited-VLN1 pathway is associated with ROS accumulation in root tips (Fig 6A and 6B).

Additionally, we examined the number of root hairs in different genotypes under ROS-inhibited drug diphenyleneiodonium (DPI) treatments [50]. DPI treatments led to a significant decrease in root hair number and the decreased level of root hair number depended on the increased DPI concentrations in Col-0, and a negative control 0.75% DMSO didn’t affect the root hair formation (Fig 6C and 6D). Further, *adf7*, *ADF7* OE, *vln1*, *VLN1* OE, *adf7 vln1*, and *adf7 VLN1* OE seedlings showed the similar number of root hairs with Col-0 under DPI treatments (Fig 6C and 6D), confirming that the function of ADF7-inhibited-VLN1 pathway on root hair formation requires ROS level elevation. RHD2 is correlated with the increased ROS levels in roots and root hairs [35–37]. Then, the *RHD2* expression was calculated in Col-0, *adf7*, *ADF7* OE, *vln1*, *VLN1* OE, *adf7 vln1*, and *adf7 VLN1* OE seedlings. The results showed that the inhibited *RHD2* expression in *adf7*, *VLN1* OE, and *adf7 VLN1* OE seedlings and the increased it in *ADF7* OE, *vln1*, and *adf7 vln1* seedlings, compared with Col-0 (Fig 6E), indicating that the role of ADF7-inhibited-VLN1 pathway in ROS level elevation might be via affecting *RHD2* expression.

### The ADF7-inhibited-VLN1 pathway is essential for root hair formation in plant osmotic stress tolerance

**Fig 7:**
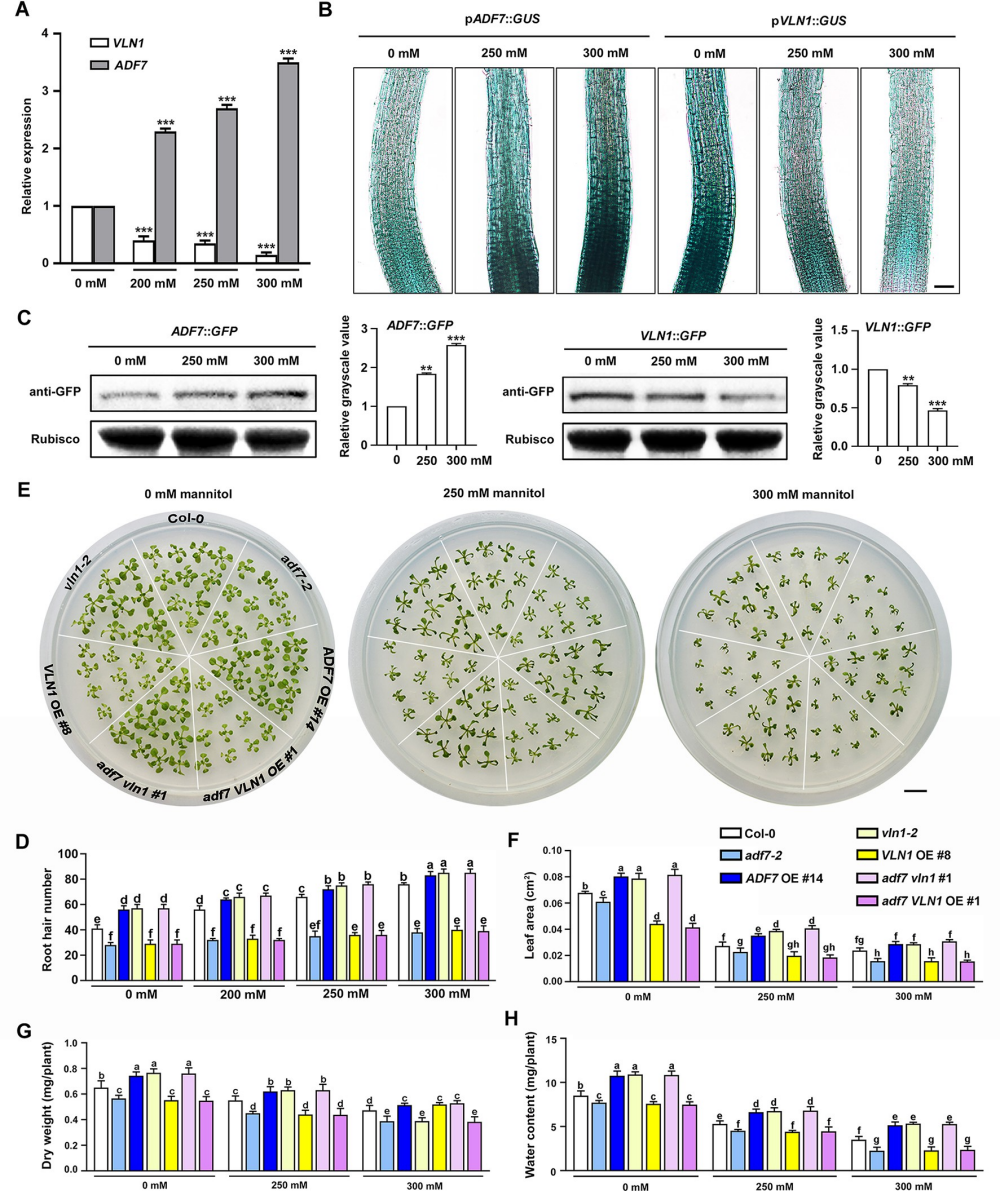
The pathway of ADF7-inhbited-VLN1 is crucial for root hair formation in plant osmotic stress tolerance. **(A)** Relative expression level of *ADF7* and *VLN1* in roots from 6-d-old Col-0 seedlings with 200, 250, and 300 mM mannitol treatments. Values are means ± SD of three independent biological replicates. *** *P*< 0.001, Student’s *t*-test compared with Col-0. **(B)** GUS activity of 4-d-old seedlings carrying the p*ADF7*::*GUS* or p*VLN1*::*GUS* reporter gene with 0, 250, and 300 mM mannitol treatments. Scale bar, 50 μm. **(C)** Western blotting using proteins extracted from different genotypes carrying the p*ADF7*::*ADF7-GFP* gene or p*VLN1*::*VLN1-GFP* gene with 0, 250, and 300 mM mannitol treatments. Rubisco was used as a loading control. Histograms depict quantification of the relative grayscale value. Values are means ± SD of three independent biological replicates. ** *P*< 0.01, *** *P* < 0.001, Student’s *t*-test compared to Col-0 with 0 mM mannitol treatment. **(D)** Histogram depicting root hair number from *ADF7* and *VLN1* genotypes under 0, 200, 250, and 300 mM mannitol treatments. **(E)** Images of growth state from *ADF7* and *VLN1* genotype seedlings under 0, 250, and 300 mM mannitol treatments. Scale bar, 1 cm. **(F) to (H)** Phenotypic characteristics of leaf area **(F)**, dry weight **(G)**, water content **(H)** from 16-d-old genotype seedlings under 0, 250, and 300 mM mannitol treatments. Significant differences (*P*< 0.05; indicated by different letters) among genotypes are determined for each condition by one-way ANOVA followed by Tukey’s test in **(D)**, **(F)**, **(G)**, and **(H)**.

Microarray data revealed that osmotic stress leads to the changes of *ADF7* and *VLN1* expression in roots [51]. We, therefore, considered whether ADF7-inhibited-VLN1-regulated root hair formation functions in plant osmotic stress tolerance. Consistent with the microarray data, RT-qPCR analysis showed that *ADF7* and *VLN1* expression in roots was increased and decreased by mannitol treatments, respectively (Fig 7A). This was further verified by Gus staining and western blot analysis (Fig 7B and 7C). Moreover, Col-0 showed the increased root hair numbers under 200 mM, 250 mM, and 300 mM mannitol treatments (Fig 7D), confirming that osmotic stress-induced root hair formation. Osmotic stress-induced root hair formation was dependent on increasing mannitol concentration, consistent with the osmotic-mediated *ADF7* and *VLN1* expression mode dependent on increasing mannitol concentration in roots (Fig 7A and 7D).

Compared with Col-0, *ADF7 OE*, *vln1*, and *adf7 vln1* seedlings grew more root hair numbers under mannitol treatments (Fig 7D). In contrast, *adf7*, *VLN OE*, and *adf7 VLN* OE seedlings displayed defects in osmotic stress-induced root hair formation (Fig 7D). Additionally, compared with Col-0, *vln1*, *ADF7 OE*, and *adf7 vln1* seedlings showed better growth state with bigger leaf area, higher dry weight, and higher water content under normal condition and osmotic stress, while *adf7*, *VLN1 OE*, and *adf7 VLN1* OE seedlings displayed contrasting results (Fig 7E–7H). These results highlighted that the root hair formation mediated by ADF7-inhibited-VLN1 contributes to water uptake and osmotic stress tolerance.

**Fig 8:**
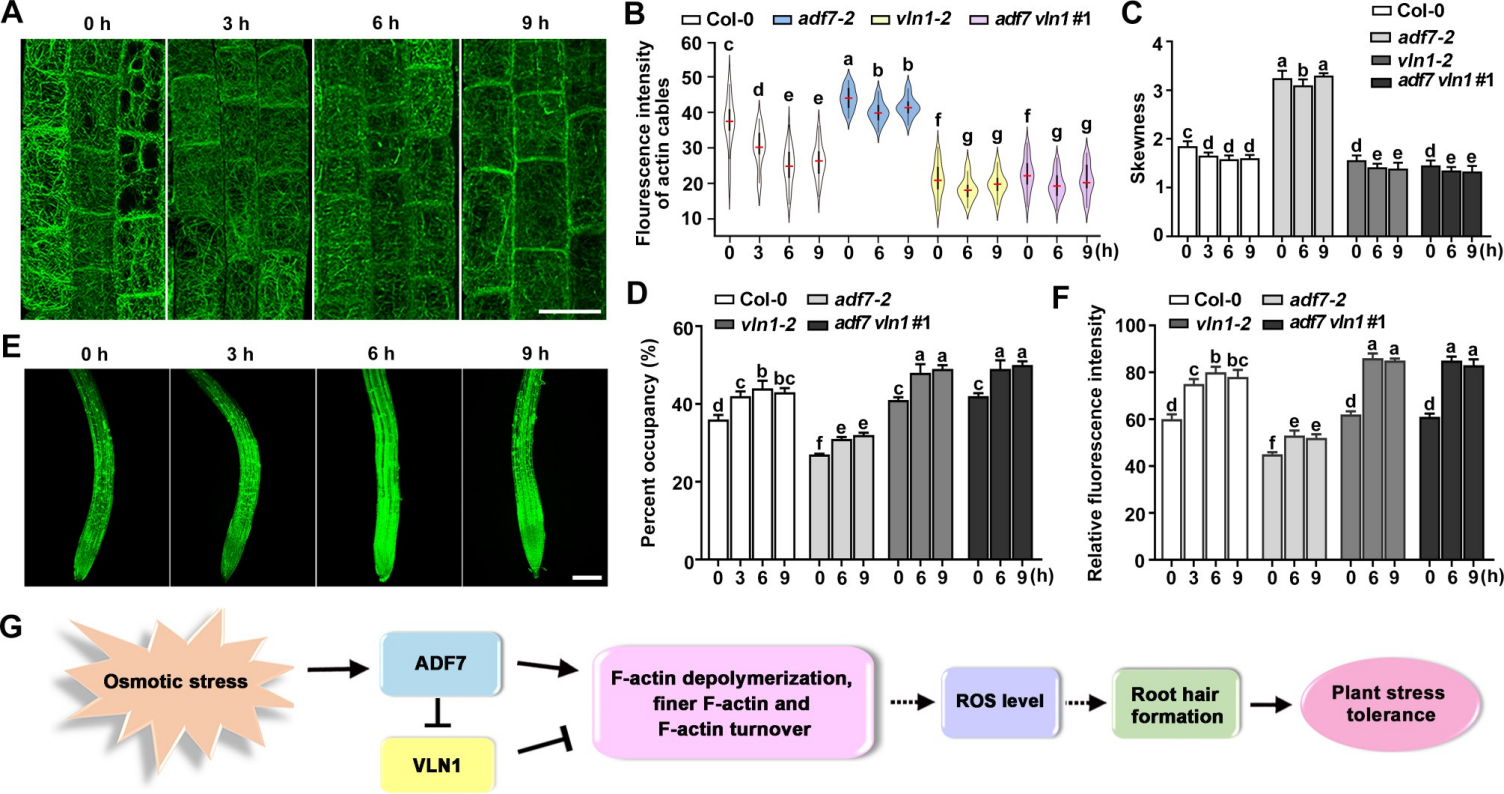
The pathway of ADF7-inhibited-VLN1 is involved in osmotic-induced F-actin depolymerization and ROS accumulation. **(A)** Confocal microscopy images of epidermal cells visualized by the expression of *fABD2*::*GFP* in root apices from Col-0 seedlings under mannitol treatments (200 mM) for 0, 3, 6, and 9 h. Scale bar, 20 μm. **(B)** Violin plot showing the average and contribution of fluorescence intensity of actin cables in different genotypes seedlings under 200 mM mannitol treatments for different times. The red line represents the average fluorescence intensity in different genotypes. **(C)** Histogram depicting skewness of F-actin in **(B)**. **(D)** Histogram depicting the percentage of occupancy of F-actin in **(B)**. **(E)** ROS production in the roots of Col-0 seedlings under mannitol treatments (200 mM) for 0, 3, 6, and 9 h. Scale bar, 100 μm. **(F)** Histogram depicting relative fluorescence intensities of the ROS levels in **(E). (G)** Working model of ADF7 and VLN1 in root hair formation and in response to osmotic stress in *Arabidopsis*. Arrows represent positive regulation, and bar ends mean inhibitory action. Osmotic stress induces ADF7 expression, which leading to the inclined F-actin depolymerization and the inhibited VLN1 expression that decreases F-actin bundle. The cooperation of ADF7 and VLN1 results in the increased F-actin depolymerization, finer F-actin and F-actin turnover, which associated with the increase ROS level and root hair formation in osmotic stress, then enhancing plant osmotic stress tolerance. At least 30 seedlings were examined for each genotype in different treatments. Significant difference (*P*< 0.05) indicated by different letters among genotypes is determined for each condition by one-way ANOVA, followed by Tukey’s test in **(B)**, **(C)**, **(D)**, and **(F)**.

Furthermore, we observed the F-actin dynamics in epidermal cells of roots from Col-0, *adf7*, *vln1*, and *adf7 vln1* under 200 mM mannitol treatments for a different time. Under mannitol treatments for 3 h, 6 h, and 9 h, F-actin depolymerization was clearly observed (Fig 8A), consistent with the decline of fluorescence intensity and skewness parameter and the increase of the percentage of occupancy (Fig 8A–8D). Among these treatments, the F-actin depolymerization was the relative most significant in 6 h; Further, *ADF7* deletion blocked the osmotic-induced F-actin depolymerization, on the contrary, *VLN1* single-gene deletion and *ADF7* and *VLN1* double gene deletion promoted it (Fig 8A–8D). These results demonstrated that osmotic-induced F-actin depolymerization was regulated by the pathway of ADF7-inhibited-VLN1. Furthermore, we observed the ROS accumulation in the above same conditions. Col-0 displayed the increase of ROS in 3 h, 6 h, and 9 h treatments, osmotic-induced ROS accumulation was blocked in *adf7* and promoted in *vln1* and *adf7 vln1*, which was associated with F-actin dynamics (Fig 8E and 8F).

Combined with the above results, we concluded that osmotic stress induces *ADF7* expression, and then the increased ADF7 promotes F-actin depolymerization and inhibits *VLN1* expression and the function of VLN1-mediated F-actin bundled. The cooperation of ADF7 and VLN1 leads to the increase of F-actin depolymerization, finer F-actin and F-actin turnover, which associated with the increased ROS level and root hair formation in osmotic stress, then enhancing plant osmotic stress tolerance (Fig 8G).

## Discussion

The actin cytoskeleton is required for root hair formation [3,9–11]. Different kinds of ABPs are involved in regulating F-actin arrays and dynamics in root hair formation [20,21]. To this day, a few *Arabidopsis* ABPs, such as CROOKED/ARPC5 and AtPRF1, are identified to function in root hair formation using genetic analysis in plants [15,20,21,52]. Therefore, the molecular mechanisms of actin cytoskeleton in root hair formation are largely unknown. Here, our results revealed novel evidence on the role and interaction of two ABPs in root hair formation. Actin depolymerization factor ADF7 and actin bundling protein VLN1 co-regulated F-actin dynamics to participate in root hair formation, which correlated with ROS accumulation in response to osmotic stress (Fig 8G).

### ADF7 inhibits VLN1 to regulate root hair formation by controlling F-actin dynamics

Previous findings showed that ADF7 plays an important role in flower development [28,53]. Our previous finding illustrates that VLN1 negatively regulates root hair growth [33]. Here, we further studied the physiological roles of ADF7 and VLN1 during root hair formation. We found that *adf7* mutants showed fewer root hairs and *ADF7* overexpression seedlings displayed more root hairs (Fig 1A–1C). More and fewer root hairs in *vln1* mutants and *VLN1* OE seedlings were observed, respectively (Fig 1D–1F). To this day, the location of any ADFs is not reported in root cells by GFP signals. Although we introduced a p*ADF7*::*ADF7*::*GFP* construct into *adf7-2*, the clear structure from GFP signals was also not observed in root cells. Fortunately, the location of VLN1 by GFP signals shows several bright filaments in roots [29], as well as that GUS staining analysis also indicates that VLN1 and ADF7 express in root tips (Fig 2C; [29,33]), supporting the function of VLN1 and ADF7 on root hair formation. Further analysis established a close relationship of ADF7 and VLN1 in root hair formation through the gene or protein expression mode, the genotype phenotypes, and F-actin dynamics (Fig 2). These results indicate that ADF7 inhibits VLN1 to regulate F-actin dynamics in root hair formation, revealing the novel physiological roles of ADF7 and VLN1 in root hair formation.

ADF7 and VLN1 can bind to G-actin *in vitro* and collocate with F-actin *in vivo*, demonstrating that ADF7 and VLN1 are actin binding proteins [24,28,29,32]. Further, it is identified that ADF7 mediates depolymerizing/severing single F-actin and VLN1 only expresses bundling F-actin activity [28,32]. Additionally, it is found that VLN1 and ADF1 may compete with F-actin *in vitro*, suggesting that VLN1 and ADFs might interact in regulating actin dynamics [28]. Our results demonstrate that ADF7 inhibits the VLN1-mediated F-actin bundling process in the epidermal cells of roots, outgrowing bugles and the new emerged root cells. To this day, all previous reports show that the deletion *ADF*s result in the significant increase of thick bundles in planta, such as *adf7* pollen tubes, *adf4* hypocotyl epidermal cells, *ADF2*-RNAi seedlings [28,54–56], which may be due to the fact that the very fast single F-actin depolymerizing and severing can cause the short F-actin and rapid disappearance of long F-actin, which doesn’t contribute to bundle two single F-actin. Therefore, it seems that there might be the same regulation mechanism that ADFs inhibit F-actin bundling process in various cells.

Our analysis of RT-qPCR, GUS staining, and western blotting illustrate that ADF7 inhibits *VLN1* gene or protein expression in root tips (Fig 2A–2F). Many previous reports support the function of ADFs in regulating gene expression; for example, AtADF4 modifies *RPS5* expression [49]; OsADFs regulate the expression of some defense-related genes [57]; and AtADF9 affects *FLC* expression [48]. It has been hypothesized that some ABPs might regulate actin to enter the nucleus in plants to regulate gene expression in plants, similar to the mechanisms in humans [58–61]. Here, our results demonstrated that ADF7 inhibited the *VLN1* expression in roots. The reason might be that ADF7 promotes F-actin depolymerization, which might activate the role of actin in regulating gene expression, leading to decreased *VLN1* expression. In addition, VLN1 competes with ADFs to bind actin *in vitro* [32]. The increase of ADF7 expression leads to enhance the amount of bound actin filaments, so less free actin filaments might require less VLN1 to bind *in vivo*. Therefore, plants might produce a signal of inhibition VLN1 according to their requirement.

VLN1 and VLN3 play a partially overlapping role in the turnover of actin bundle formation *in vitro* [34]. We also found that the expression of *VLN3* in *adf7-2* and *ADF7* comp #2, *ADF7* comp #7, *ADF7* OE #13, and *ADF7* OE #14 was similar, suggesting that ADF7 might not affect other VLN family factors, such as VLN3 (S8 Fig). In addition, we found that ADF7 didn’t significantly regulate *VLN1* expression in leaves by the analysis of RT-qPCR and GUS staining (S9 Fig). The reason might be that no detectable expression of *ADF7* in leaves leads to no or weak function of ADF7 in leaves [24].

Consistent with the previous results, our observation of F-actin dynamics in Col-0 root epidermal cells in the root hair emission region showed that numerous longitudinal F-actin (parallel growth axes) surrounded the nuclei near the end walls (S10 Fig, [12]). Our results further revealed that the assembled longitudinal F-actin existed ahead of the root hair growth site in Col-0, which might provide the original actin with root hairs for outgrowing. The double mutant *act2 act7* displays defects in root hair formation [10]. In swollen root epidermal cells of *act2 act7*, the fewer, thick, and transversely oriented F-actin bundles or rod-like structures instead of the more, fine, and longitudinal F-actin in that of WT seedlings [10]. This indicates that the orientation, thickness, and density of F-actin in root epidermal cells are closely related to root hair formation [3,9–11].

We observed the F-actin dynamics in root epidermal cells in the transition, elongation/differentiation, and root hair regions, as well as the outgrowing bugles and new emerged root hairs in cross or longitudinal sections in the *ADF7* and *VLN1* genotype seedlings. Our results found that ADF7 and VLN1 significantly affected the thickness and density of F-actin (Fig 3). ADF7 loss-of-function led to the thicker bundles and less amount of F-actin, associated with the declined depolymerizing rate, severing frequency, and F-actin turnover (Fig 4). While VLN1 loss-of-function display finer bundles and more amount of F-actin, associated with the declined single F-actin-bundling frequency and the increased depolymerizing rate, severing frequency, and F-actin turnover (Fig 4). These F-actin arrays and dynamics are consistent with the biochemistry properties of ADF7 and VLN1 and further support the notion of the thickness and density of F-actin are closely related to the root hair formation [3,9–11,28,32]. Further, genetic analysis and pharmacological experiments found that the finer and more F-actin and increased F-actin turnover regulated by ADF7-inhibited-VLN1 pathway in root hair formation (Fig 5). Some previous findings suggested the connection between F-actin arrays and root hair formation. For example, PRF1 contributes to F-actin assembly, and *prf1* shows more root hairs, suggesting that the disassembled F-actin actives root hair formation [15]. The phenotypes of *act2 act7* demonstrate that the thicker and fewer F-actin in the root epidermal cells of the root hair emission region is close to the root hair formation inhibition [10]. Our results demonstrate that ADF7-inhibited-VLN1 directly regulates F-actin arrays and dynamics by promoting mild depolymerization, inhibiting thick bundles, and forming more fine bundles to active root hair formation.

### ADF7-inhibited-VLN1-regulated F-actin dynamics are involved in ROS accumulation and plant osmotic stress response

ROS signaling pathway plays an important role in root hair formation and in response to abiotic stress [35–37,62]. Our results showed that increased ROS level was found in the seedlings with more root hairs and mild F-actin depolymerization, including *ADF7* OE, *vln1*, and *adf7 vln1* (Fig 6A and 6B); by contrast, the decreased ROS level was found in the seedlings with fewer root hairs and F-actin stabilization, including *adf7*, *VLN1* OE, and *adf7 VLN1* OE seedlings (Fig 6A and 6B); Lat A application with low concentrations activated ROS accumulation and root hair formation (Fig 6A and 6B); DPI treatments inhibited ADF7-inhibited-VLN1-regulated root hair formation (Fig 6C and 6D); ADF7-inhibited-VLN1 decreased the *RHD2* expression (Fig 6E). These results demonstrate that the mild F-actin depolymerization precedes ROS accumulation during root hair formation, whose behavior is also observed by several pieces of previous evidence that mild F-actin depolymerization elevate ROS level *in vivo* [26,39,40]. RHD2 positively regulates root hair formation by elevating ROS accumulation [35–37]. It has been reported that F-actin depolymerization increases *RHD2* expression to elevates ROS levels in salt stress in *Arabidopsis* [39]. Therefore, our results suggest that ADF7-inhibited-VLN1 regulates mild F-actin depolymerization and F-actin turnover, which associated with the increasing *RHD2* expression and ROS level in root hair formation.

It is well accepted that water deficiency-induced root hair development plays a beneficial attribute for maximizing water absorption to enhance plant stress tolerance [7,8]. Gene expression analysis reveals that numerous genes are involved in root hair development under water deficiency [7]; Water deficiency is caused by a series of environmental factors, such as osmotic stress and drought stress [7,63]. However, the molecular mechanisms of root hair formation in osmotic stress were largely unknown, and only several proteins, such as MaRHD3, OsWOX11, EVP1, OsGH3-2, involved in drought-induced root hair initiation are identified using genetic analysis [57,64–66]. However, the role of actin cytoskeleton in root hair initiation responses to osmotic stress was largely unknown. It is well acceptable that the increased ROS accumulation responds to abiotic stress, such as osmotic stress, salt stress, or drought [62]. Our results indicate that F-actin depolymerization regulated by ADF7-inhibited-VLN1 is involved in promoting ROS accumulation in root hair formation in osmotic stress, which plays an important role in plant tolerance osmotic stress, providing novel evidence on the molecular mechanisms of actin dynamics in response to abiotic stress.

Actin cytoskeleton is required for cell growth, cell difference and cell signal transduction [61,67]. Actin dynamics are complex and well-organized subtle changes from the interaction of various single F-actin dynamics. Investigating the molecular interaction of various single F-actin dynamics in responses to environmental stimuli is the scientific challenge. Here, we provide the novel single F-actin dynamics and their molecular mechanisms in plant physiological activities, that ADF7 inhibits VLN1-organised thick bundles to increase F-actin depolymerization, fine F-actin and F-actin turnover, associated with root hair formation and plant osmotic stress tolerance.

## Methods

### Plant growth and treatment conditions

All the seedlings were the Columbia ecotype in this study. The mutants used in this study were listed as follows: *adf7-2* (Salk_024537), *vln1-1* (Salk_020027), and *vln1-2* (Salk_133579). *Arabidopsis* seeds were sterilized and plated on 1/2MS medium with 0.8% (w/v) agar (pH 5.8). The *Arabidopsis* plants were grown in a growth chamber at 22°C under a 16 h light and 8 h darkness photoperiod. For ET pharmacological treatment, 3-d-old seedlings were transferred to 1/2 MS medium with EHT or 1-methylcyclopropene (1-MCP) added and then vertically grew for 2 d. For leaf area, dry weight, water content, 3-d-old seedlings were removed to 0 mM, 250 mM, and 300 mM mannitol media to grow for 13 d, then the 16-d old seedlings were tested at least 60 plants with three technical and biological replicates. Leaf area was the average of all leaves per plant. Dry weight of the seedlings was measured after a 16 h at 80°C oven treatment. Water content was calculated as follows: (fresh weight-dry weight)/plant.

### Plasmid construction and plant transformation

For the p*ADF7*::*GUS*, the *ADF7* promoter fragment was inserted into binary vector *pCAMBIA1300-221* using unique PstI and SmaI restriction sites. *ADF7* promoter and *ADF7* CDS fragment were cloned to generate the p*ADF7*::*ADF7*::*GFP* construct in the p*Super1300*. Primers are listed in S1 Table. Plasmids were transformed into *Agrobacterium tumefaciens* GV3101 and introduced into *Arabidopsis* Col-0 using the floral dip method. Three generations of transgenic plants were selected on 1/2 MS medium containing 30 μg/mL hygromycin until homozygous material was obtained.

### Root hair number analysis

Root hair number was calculated including root hairs and bulges on the visible side in a distant between 2 and 4 mm from the primary root tips, as the previous method [4]. To measure the percentage of root hairs from H or N cells, we, respectively, calculated the proportion of root hairs from five adjacent H or N cell files in the fixed zone between 2.5 and 3.5 mm from the primary root tips from 20 roots for each genotype (total of 100 cells per genotype), as the previous method [41]. To measure root hair formation portion in H cells per mm, we calculated the number of root hairs from a line of H cells in the fixed zone between 2.5 and 3.5 mm from the primary root tips from 20 roots for each genotype (total of 100 cells per genotype), as the previous method [42]. For actin pharmacological experiments, the 3-d-old seedlings were treated with the presence or absence of actin disrupting drug Lat A with 0.2 and 0.4 μM for 3 h and washed out the drugs. Then, the seedlings were removed to normal 1/2 MS media to grow for 3 d for calculating root hair numbers from at least 50 roots per genotype. Image J was used to measure root hair numbers in the same focal plane.

### ROS level analysis

ROS was observed in the root tips of 3-d-old seedlings by H2DCF–DA (2′,7′-dichlorodihydro-fluorescein diacetate) as the previous method [37]. The images between 0 and 1mm from root tips were collected by laser scanning confocal microscope (Nikon) using a ×10 objective with the 488-nm laser. Within experiments, gain, pinhole, laser power, and detector offset were set to the same parameters. Experiments were repeated at least three times. ROS intensity of root was measured in >30 individual seedlings per genotype respectively used Image J.

### RNA isolation and gene expression analysis

RNA extraction buffer was from the Easy Pure Plant RNA kit (TRANS). Quantitative PCR with reverse transcription (RT-qPCR) was performed using Bio-rad CFX96. Primers used are listed in (S1 Table). The histochemical GUS staining assay was performed using 4-day-old p*VLN1*::GUS, p*ADF7*::GUS transgenic seedlings and hybrid materials in a 37°C incubator for 3 h. After decolorization with ethanol and acetic acid solutions, the images were taken by Upright microscope (Nikon model eclipse Ni-U) with Nikon DS-Ri2 Microscopic imaging system through 20 times objective lens.

### Western blotting assays

Protein was extracted from 10-day-old seedlings which carrying the *VLN1*::*GFP* gene and *ADF7*::*GFP* gene. The protein was analyzed by SDS-PAGE. An anti-GFP antibody (Thermo Fisher) contained a dilution of 1:30,000 in TBST (50 mM Tris, 150 mM NaCl, and 0.05% (v/v) Tween 20, pH 7.5) was used as a probe, a dilution of 1:10,000 in a rabbit anti-mouse IgG H&L (HRP) secondary antibody (Abcam). The bands were detected by Hypersensitive ECL Chemiluminescence Kit. Rubisco bands were used as loading controls.

### Quantitative analysis of F-actin arrays

Actin filament architecture was quantitatively analyzed using three parameters including average and contribution of GFP fluorescence intensity of actin cables, percentage of signal occupancy (density), and skewness [45,46]. The F-actin in every root cell from a fixed zone in the transition zone and the elongation/differentiation zone [43]. GFP was excited by a 488-nm laser. A fixed laser power and gain setting were used for different genotypes. For statistical analysis, we measured fluorescence intensity, skewness, and density values in at least 60 images of root cells from at least 20 individual seedlings in every genotype.

### Time-lapse imaging of signal F-actin dynamics

The time-lapse imaging of actin filament dynamics in living cells was performed using the general method [45]. Briefly, surface-sterilized seeds were sown on a coverslip on one-half strength MS medium and the coverslip was tilted in a Petri dish, then the Petri dish was placed horizontally for 4 d. micrographs of actin filaments were collected every 3 s by laser scanning confocal microscope (Nikon) using a 100 × oil immersion objective. Slice thickness was 0.5 μm. The parameter settings of gain, laser power, and detector offset were the same in all experiments. As in the previous reports, the filamentous structures with smaller intensity values form a population and are assumed to be single filaments [28,46,55]. The parameters were counted by Image J. We only count filaments surviving for at least 10 s and longer than 2 μm. The maximum filament length was defined as the longest length of the tracked filament during its growth. Maximum filament lifetime was determined by the time of the tracked filament from its appearance to disappearance. Severing frequency indicates the number of breaks per unit length per unit time (break/μm/s). The severing frequency was counted from the filament at maximum length to its disappearance. Depolymerization rate was counted as changes of length in unit time (△length/△time). The bundling frequency was defined as the bundling events per unit area unit time (events/μm^2^/s). In root cell, a 30×30 -μm^2^ region was selected, At least 60 root cells from at least 20 individual seedlings are calculated in every genotype.

### Statistical analysis

Violin plots depicted the average value and the distribution of fluorescence intensity of actin cables in different genotypes or under different treatments using the methods described previously [68]. Other data was calculated by One-way ANOVA with a post-hoc Tukey and least significant difference (LSD) test on a significant level of *P* < 0.05 or by Student’s *t*-test (* *P*< 0.05, ** *P*< 0.01, *** *P*< 0.001).

## Supporting information

S1 Fig Molecular identification of *ADF7* genotypes. **(A)** Locations of T-DNA insertion alleles *adf7-2* (SALK_024537). Black boxes represent exons, and horizontal lines represent introns. T-DNA inserts (arrowheads) are drawn to scale. **(B)** to **(D)**, *ADF7* overexpression (*ADF7* OE) lines **(B)**, and complementation (*ADF7* comp) lines **(C)**, RT-PCR analysis in *adf7* mutants **(D)**, with *18S* used as an internal control. **(E)** RT-qPCR quantification of *ADF7* expression level in Col-0, *adf7-2*, *ADF7* comp #2, *ADF7* comp #7, *ADF7* OE #13, and *ADF7* OE #14 plants, with *18S* used as an internal control, and *ADF7* expression in Col-0 is normalized to 1. Values are means ± SD of three independent biological replicates. * *P*< 0.05, ** *P*< 0.01, *** *P*< 0.001, Student’s *t*-test compared to Col-0.
(TIF)

S2 Fig Enlarged images of root hair phenotypes of *ADF7* and *VLN1* genotypes. Enlarged images of root hair of Col-0, *adf7-2*, *ADF7* OE #14, *vln1-2*, *VLN1* OE #8, *adf7 vln1* #1, and *adf7 VLN1* OE #1. Scale bar, 200 μm.
(TIF)

S3 Fig Molecular identification of *VLN1* genotypes. **(A)** Locations of T-DNA insertion alleles *vln1-1* (SALK_020027) and *vln1-2* (SALK_133579). Black boxes represent exons, and horizontal lines represent introns. T-DNA inserts (arrowheads) are drawn to scale. **(B)** to **(D)**, *VLN1* overexpression (*VLN1* OE) lines **(B)**, and complementation (VLN1 comp) lines **(C)**, RT-PCR analysis in *VLN1* T-DNA insertional mutants **(D)**, with *18S* used as an internal control. **(E)** RT-qPCR quantification of *VLN1* expression level in Col-0, *vln1-1*, *vln1-2*, *VLN1* comp #9, *VLN1* comp #14, *VLN1* OE #7, and *VLN1* OE #8 plants, with *18S* used as an internal control, and *VLN1* expression in Col-0 is normalized to 1. Values are means ± SD of three independent biological replicates. * *P*< 0.05, ** *P*< 0.01, *** *P*< 0.001, Student’s *t*-test, compared to Col-0.
(TIF)

S4 Fig p*ADF7*::*ADF7*::*GFP* is functional. **(A)** Images of root hairs from Col-0, *adf7-2*, p*ADF7*::*ADF7*::*GFP* in *adf7-2* seedlings. Scale bar, 200 μm. **(B)** Histogram displaying root hair number in Col-0, *adf7-2*, p*ADF7*::*ADF7*::*GFP* in *adf7-2* seedlings. Values are means ± SD of three independent biological replicates. * *P*< 0.05, ** *P*< 0.01, *** *P*< 0.001, Student’s *t*-test compared to Col-0. **(C)** RT-PCR analysis of *ADF7* expression level in the seedlings of p*ADF7*::*ADF7*::*GFP* in *adf7-2*.
(TIF)

S5 Fig p*VLN1*::*VLN1*::*GFP* is functional. **(A)** Images of root hairs from Col-0, *vln1-2*, p*VLN1*::*VLN1*::*GFP* in *vln1-2* seedlings. Scale bar, 200 μm. **(B)** Histogram displaying root hair number in Col-0, *vln1-2*, p*VLN1*::*VLN1*::*GFP* in *vln1-2* seedlings. Values are means ± SD of three independent biological replicates. * *P*< 0.05, ** *P*< 0.01, *** *P*< 0.001, Student’s *t*-test compared to Col-0. **(C)** RT-PCR analysis of *VLN1* expression level in the seedlings of p*VLN1*::*VLN1*::*GFP* in *vln1-2*.
(TIF)

S6 Fig Molecular identification of *ADF7* and *VLN1* double gene genotypes. RT-PCR analysis of *ADF7* and *VLN1* gene expression in Col-0, *adf7 vln1* #1, *adf7 vln1* #2, *adf7 VLN1* OE #1, and *adf7 VLN1* OE #2 seedlings, with *18S* used as an internal control.
(TIF)

S7 Fig Lat A treatments activate *ADF7* and *VLN1*-comediated root hair formation. Histogram displaying root hair number from Col-0, *adf7-2*, *ADF7* OE #14, *vln1-2*, *VLN1* OE #8, and *adf7 vln1* #1 under CK and Lat A treatments (0.4 μM). A significant difference (*P*< 0.05) indicated by different letters among genotypes is determined for each condition by one-way ANOVA followed by Tukey’s test.
(TIF)

S8 Fig RT-qPCR quantification of *VLN3* expression level in Col-0, *adf7-2*, *ADF7* comp #2, *ADF7* comp #7, *ADF7* OE #13, and *ADF7* OE #14 seedlings. (TIF)

S9 Fig The expression of *VLN1* in leaves from Col-0, *adf7-2*, *ADF7* comp #2, and *ADF7* OE #14 seedlings. **(A)** RT-qPCR quantification of *VLN1* expression level in leaves of 6-d-old seedlings from Col-0, *adf7-2*, *ADF7* comp #2, and *ADF7* OE #14. **(B)** *GUS* analysis of *VLN1* expression from Col-0 and *adf7-2* seedlings. Scale bar, 0.25 cm.
(TIF)

S10 Fig Confocal microscopy images of *fABD2*::*GFP* in nuclei and F-actin of root epidermal cells. GFP shows the distribution of actin F-actin of root epidermal cells in the elongation/differentiation and transition regions, and the position of the nucleus was observed after 3 min of PI staining. Scale bar, 10 μm.
(TIF)

S1 Table Primers used in this study. (DOCX)

S1 Video Depolymerizing process of single F-actin in Col-0 root cells. (AVI)

S2 Video Depolymerizing process of single F-actin in *adf7-2* root cells. (AVI)

S3 Video Severing process of single F-actin in Col-0 root cells. (AVI)

S4 Video Severing process of single F-actin in *adf7-2* root cells. (AVI)

S5 Video Bundling process of single F-actin in Col-0 root cells. (AVI)

S6 Video Bundling process of single F-actin in *adf7-2* root cells. (AVI)

S7 Video Bundling process of single F-actin in *vln1-2* root cells. (AVI)

S8 Video Bundling process of single F-actin in *adf7 vln1* #1 root cells. (AVI)
