# Supplementary figures and images for: Actin depolymerizing factor ADF7 inhibits actin bundling protein VILLIN1 to regulate root hair formation in response to osmotic stress in Arabidopsis
Source: PLoS Genet. 2022 Sep 12;18(9):e1010338. doi: 10.1371/journal.pgen.1010338 (PMC9499291; doi:10.1371/journal.pgen.1010338)

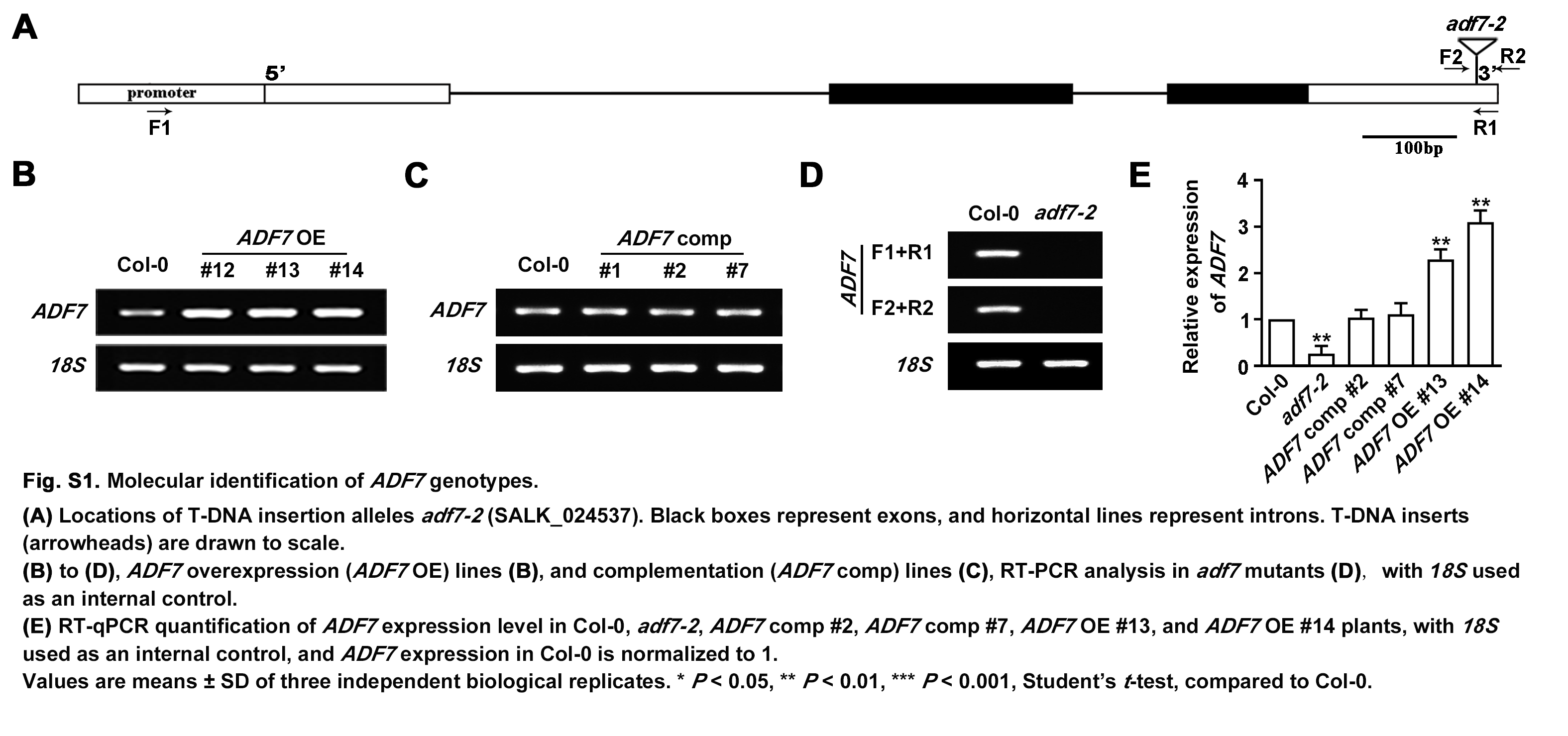

Supplement: S1 Fig — (A) Locations of T-DNA insertion alleles adf7-2 (SALK_024537). Black boxes represent exons, and horizontal lines represent introns. T-DNA inserts (arrowheads) are drawn to scale. (B) to (D), ADF7 overexpression (ADF7 OE) lines (B), and complementation (ADF7 comp) lines (C), RT-PCR analysis in adf7 mutants (D), with 18S used as an internal control. (E) RT-qPCR quantification of ADF7 expression level in Col-0, adf7-2, ADF7 comp #2, ADF7 comp #7, ADF7 OE #13, and ADF7 OE #14 plants, with 18S used as an internal control, and ADF7 expression in Col-0 is normalized to 1. Values are means ± SD of three independent biological replicates. * P< 0.05, ** P< 0.01, *** P< 0.001, Student’s t-test compared to Col-0. (TIF) [file pgen.1010338.s001.tif]

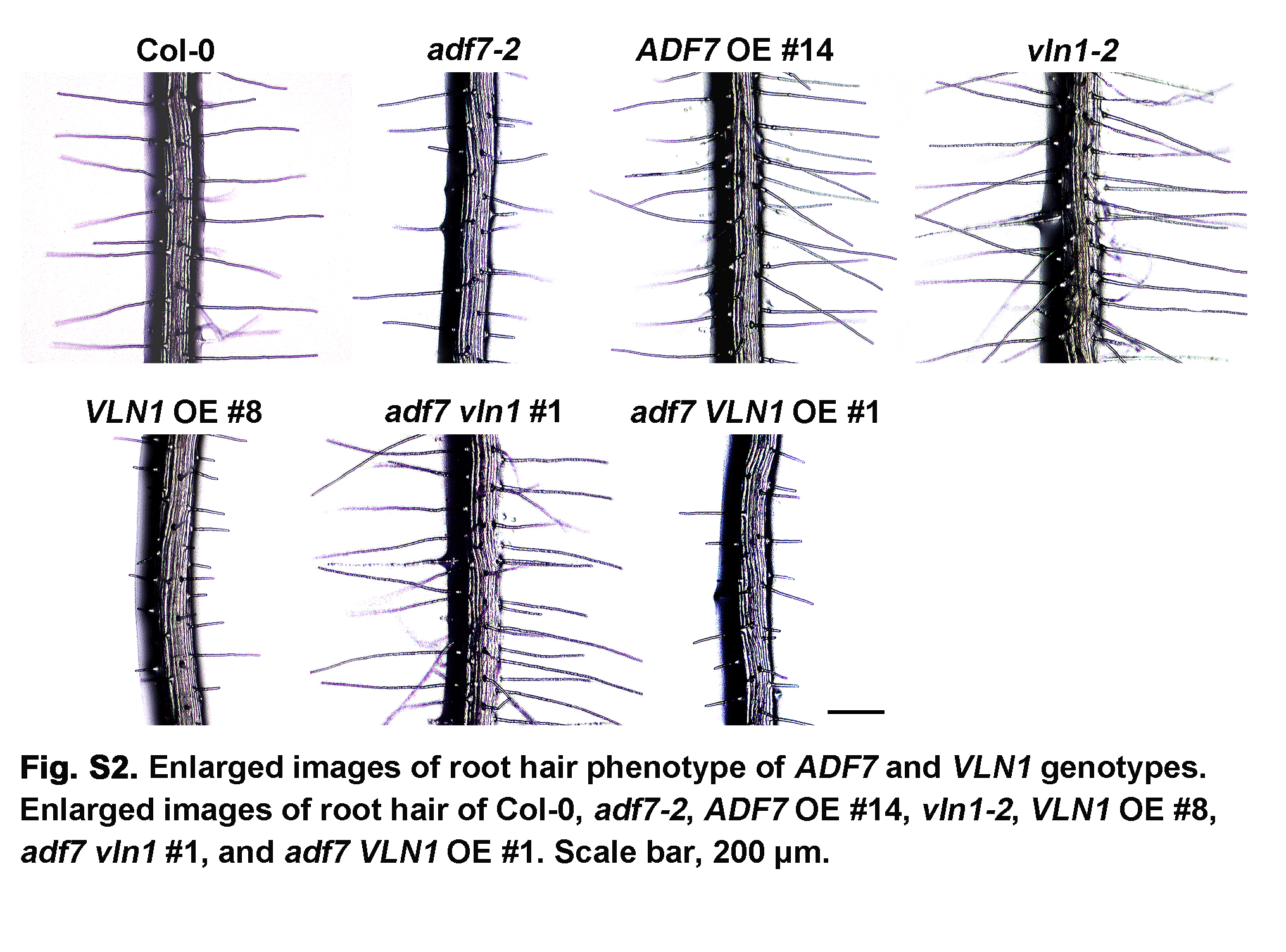

Supplement: S2 Fig — Enlarged images of root hair of Col-0, adf7-2, ADF7 OE #14, vln1-2, VLN1 OE #8, adf7 vln1 #1, and adf7 VLN1 OE #1. Scale bar, 200 μm. (TIF) [file pgen.1010338.s002.tif]

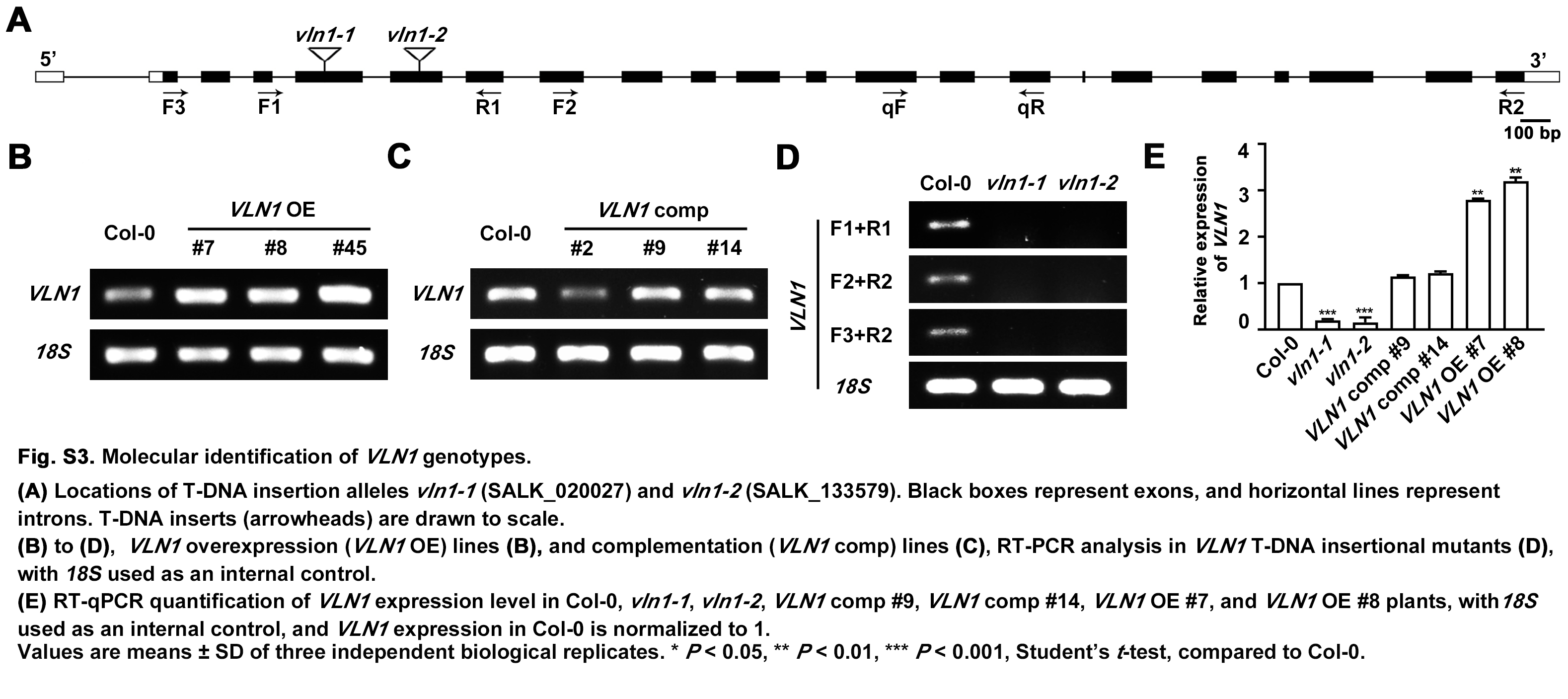

Supplement: S3 Fig — (A) Locations of T-DNA insertion alleles vln1-1 (SALK_020027) and vln1-2 (SALK_133579). Black boxes represent exons, and horizontal lines represent introns. T-DNA inserts (arrowheads) are drawn to scale. (B) to (D), VLN1 overexpression (VLN1 OE) lines (B), and complementation (VLN1 comp) lines (C), RT-PCR analysis in VLN1 T-DNA insertional mutants (D), with 18S used as an internal control. (E) RT-qPCR quantification of VLN1 expression level in Col-0, vln1-1, vln1-2, VLN1 comp #9, VLN1 comp #14, VLN1 OE #7, and VLN1 OE #8 plants, with 18S used as an internal control, and VLN1 expression in Col-0 is normalized to 1. Values are means ± SD of three independent biological replicates. * P< 0.05, ** P< 0.01, *** P< 0.001, Student’s t-test, compared to Col-0. (TIF) [file pgen.1010338.s003.tif]

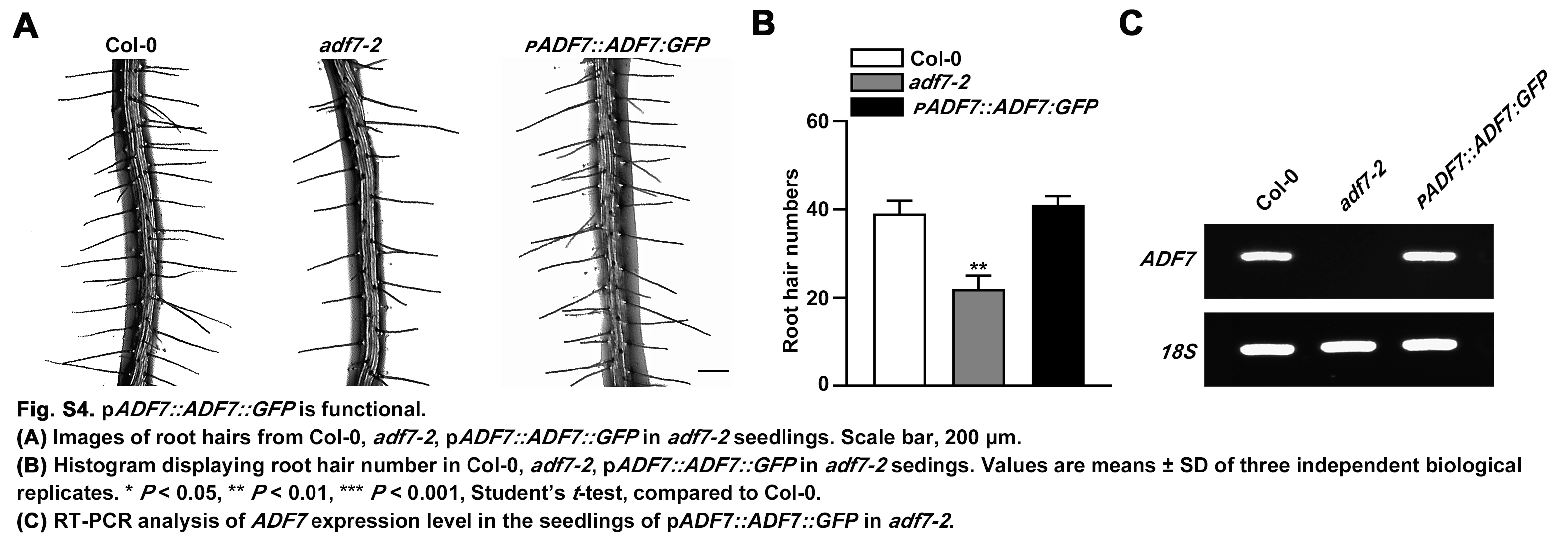

Supplement: S4 Fig — (A) Images of root hairs from Col-0, adf7-2, pADF7::ADF7::GFP in adf7-2 seedlings. Scale bar, 200 μm. (B) Histogram displaying root hair number in Col-0, adf7-2, pADF7::ADF7::GFP in adf7-2 seedlings. Values are means ± SD of three independent biological replicates. * P< 0.05, ** P< 0.01, *** P< 0.001, Student’s t-test compared to Col-0. (C) RT-PCR analysis of ADF7 expression level in the seedlings of pADF7::ADF7::GFP in adf7-2. (TIF) [file pgen.1010338.s004.tif]

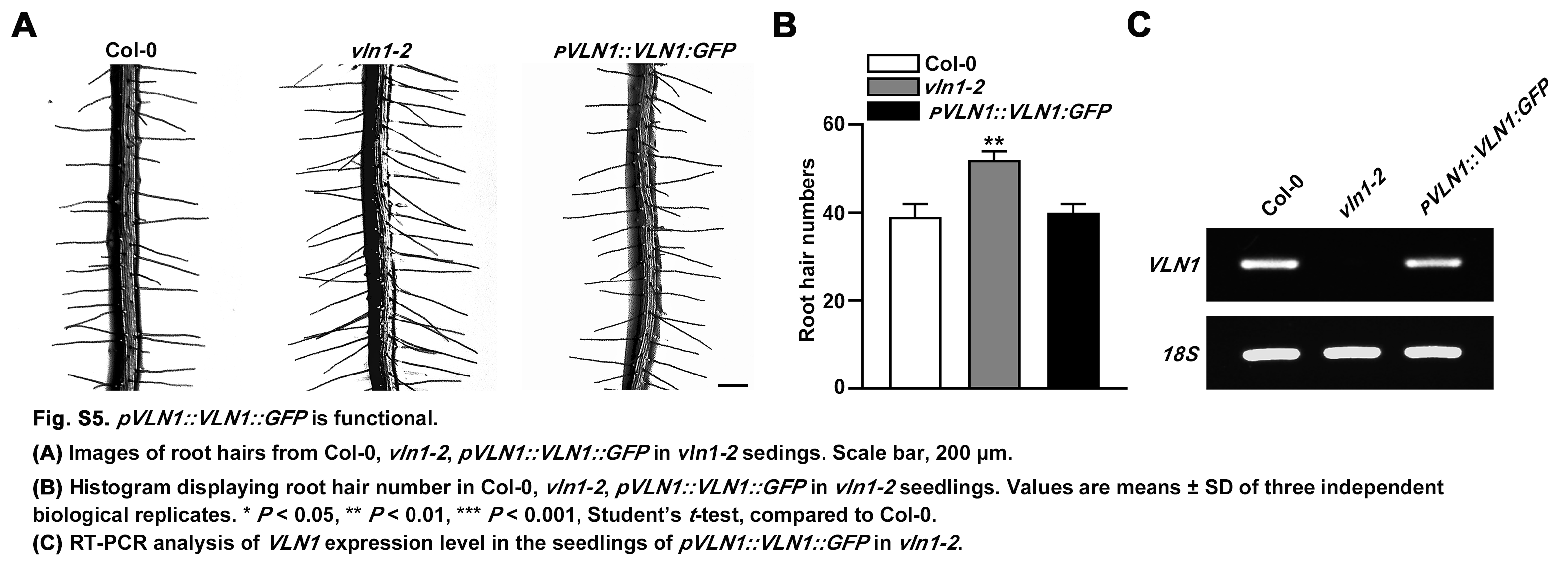

Supplement: S5 Fig — (A) Images of root hairs from Col-0, vln1-2, pVLN1::VLN1::GFP in vln1-2 seedlings. Scale bar, 200 μm. (B) Histogram displaying root hair number in Col-0, vln1-2, pVLN1::VLN1::GFP in vln1-2 seedlings. Values are means ± SD of three independent biological replicates. * P< 0.05, ** P< 0.01, *** P< 0.001, Student’s t-test compared to Col-0. (C) RT-PCR analysis of VLN1 expression level in the seedlings of pVLN1::VLN1::GFP in vln1-2. (TIF) [file pgen.1010338.s005.tif]

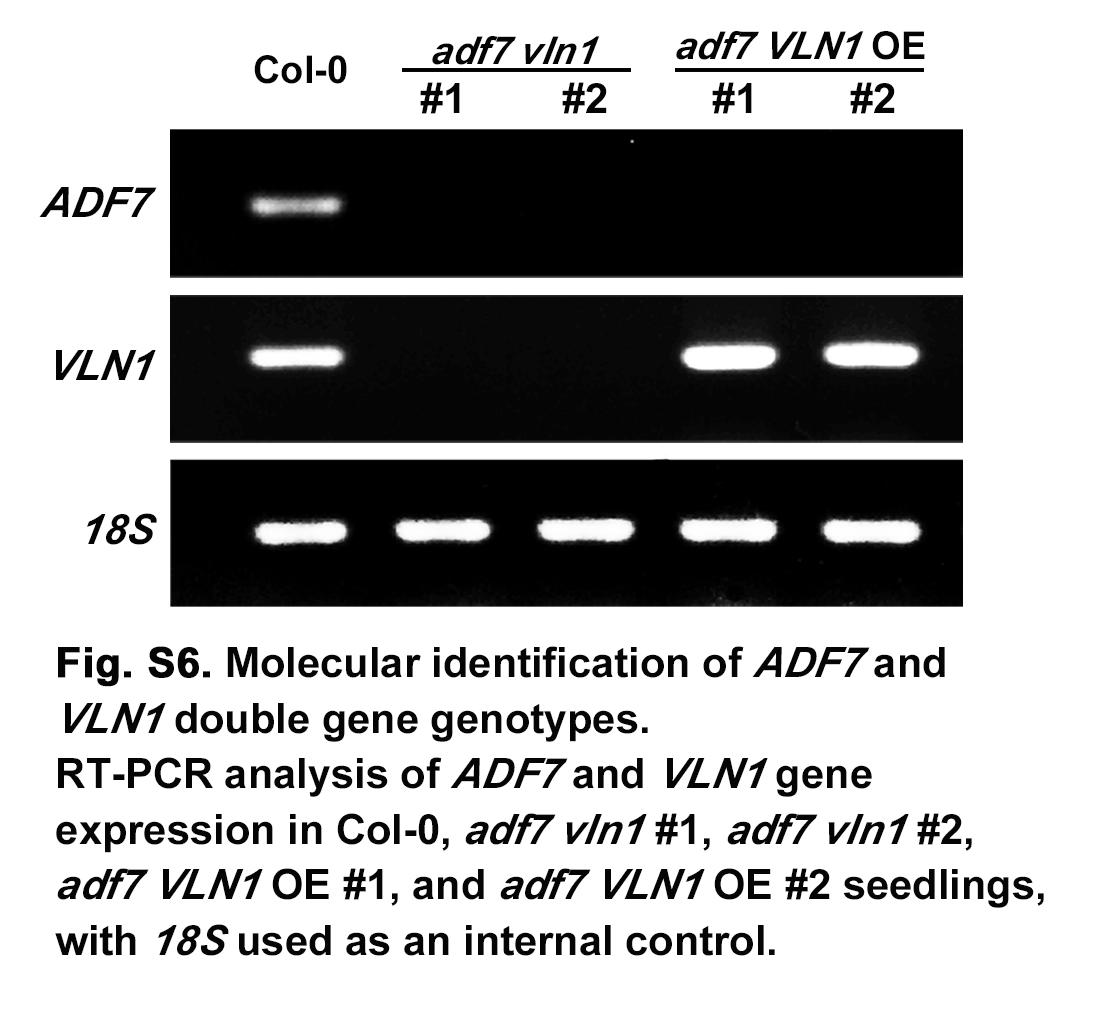

Supplement: S6 Fig — RT-PCR analysis of ADF7 and VLN1 gene expression in Col-0, adf7 vln1 #1, adf7 vln1 #2, adf7 VLN1 OE #1, and adf7 VLN1 OE #2 seedlings, with 18S used as an internal control. (TIF) [file pgen.1010338.s006.tif]

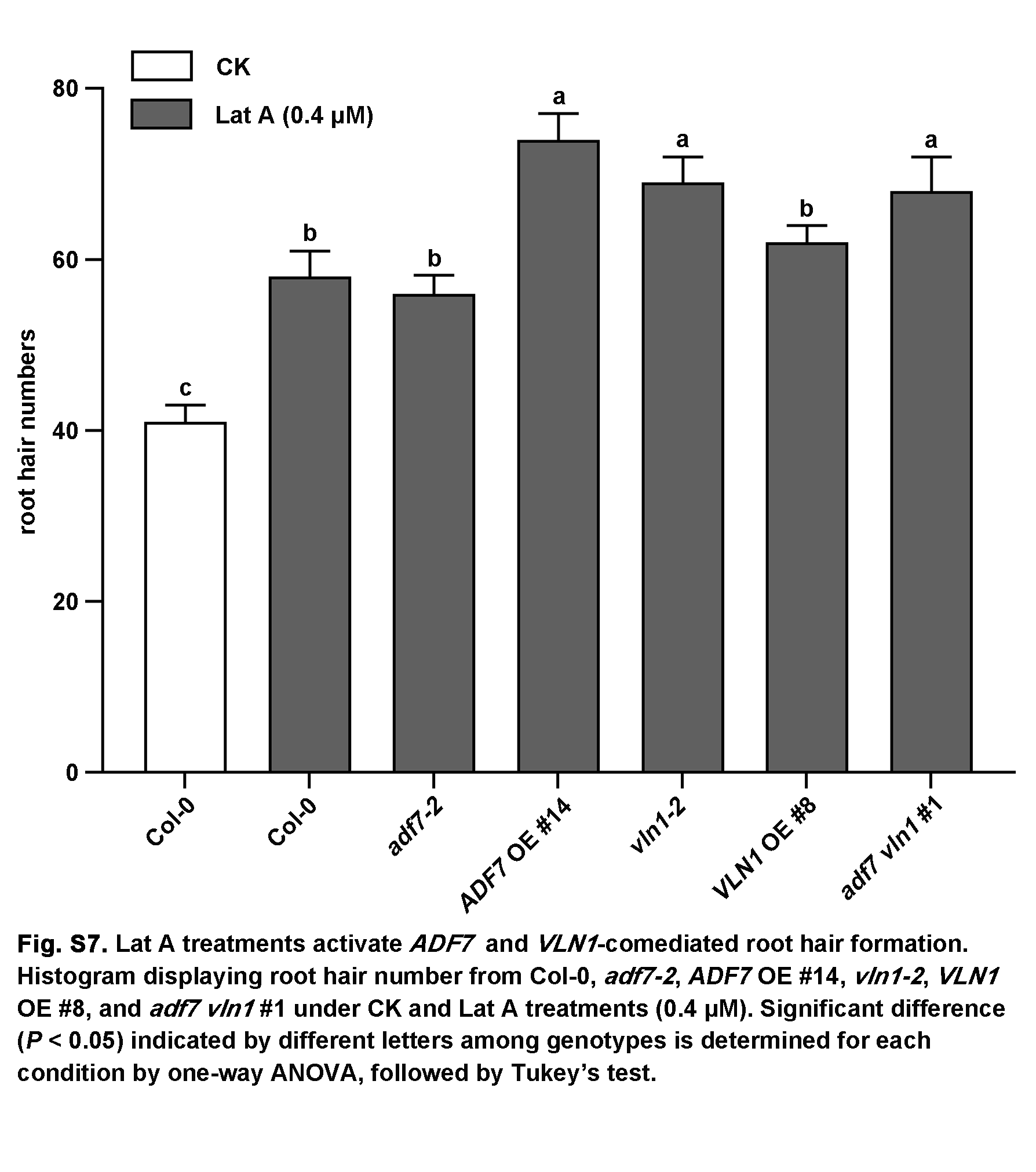

Supplement: S7 Fig — Histogram displaying root hair number from Col-0, adf7-2, ADF7 OE #14, vln1-2, VLN1 OE #8, and adf7 vln1 #1 under CK and Lat A treatments (0.4 μM). A significant difference (P< 0.05) indicated by different letters among genotypes is determined for each condition by one-way ANOVA followed by Tukey’s test. (TIF) [file pgen.1010338.s007.tif]

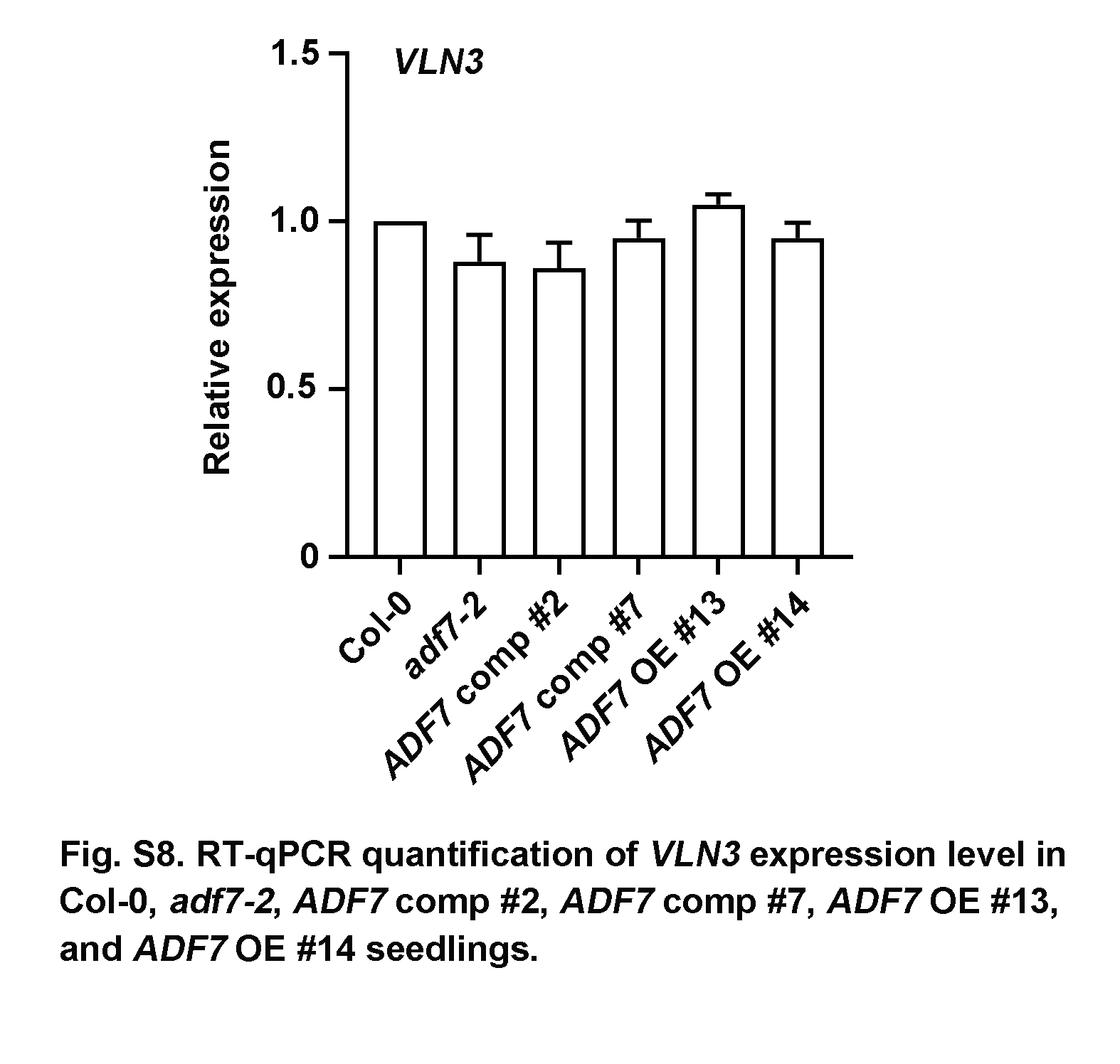

Supplement: S8 Fig — (TIF) [file pgen.1010338.s008.tif]

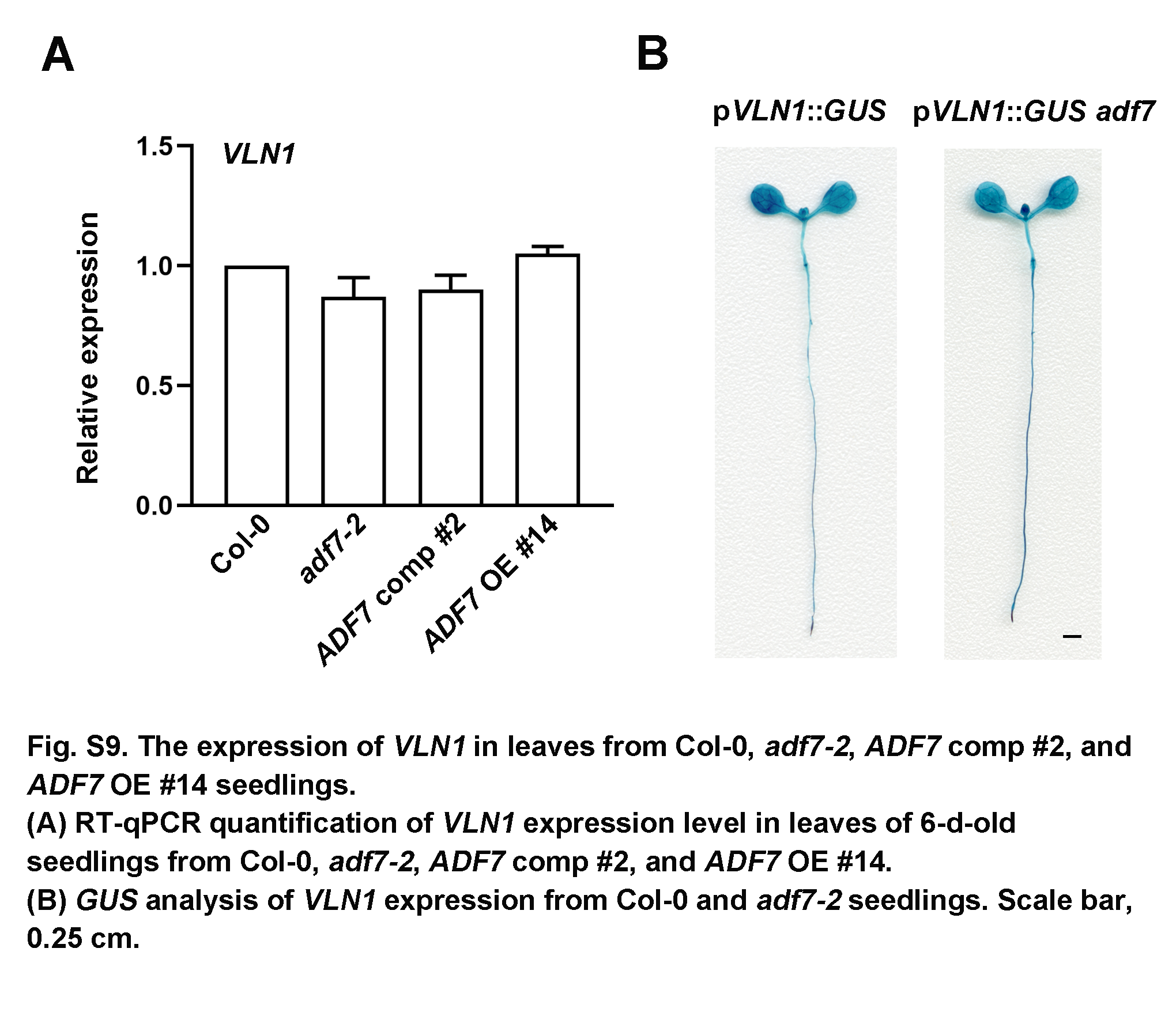

Supplement: S9 Fig — (A) RT-qPCR quantification of VLN1 expression level in leaves of 6-d-old seedlings from Col-0, adf7-2, ADF7 comp #2, and ADF7 OE #14. (B) GUS analysis of VLN1 expression from Col-0 and adf7-2 seedlings. Scale bar, 0.25 cm. (TIF) [file pgen.1010338.s009.tif]

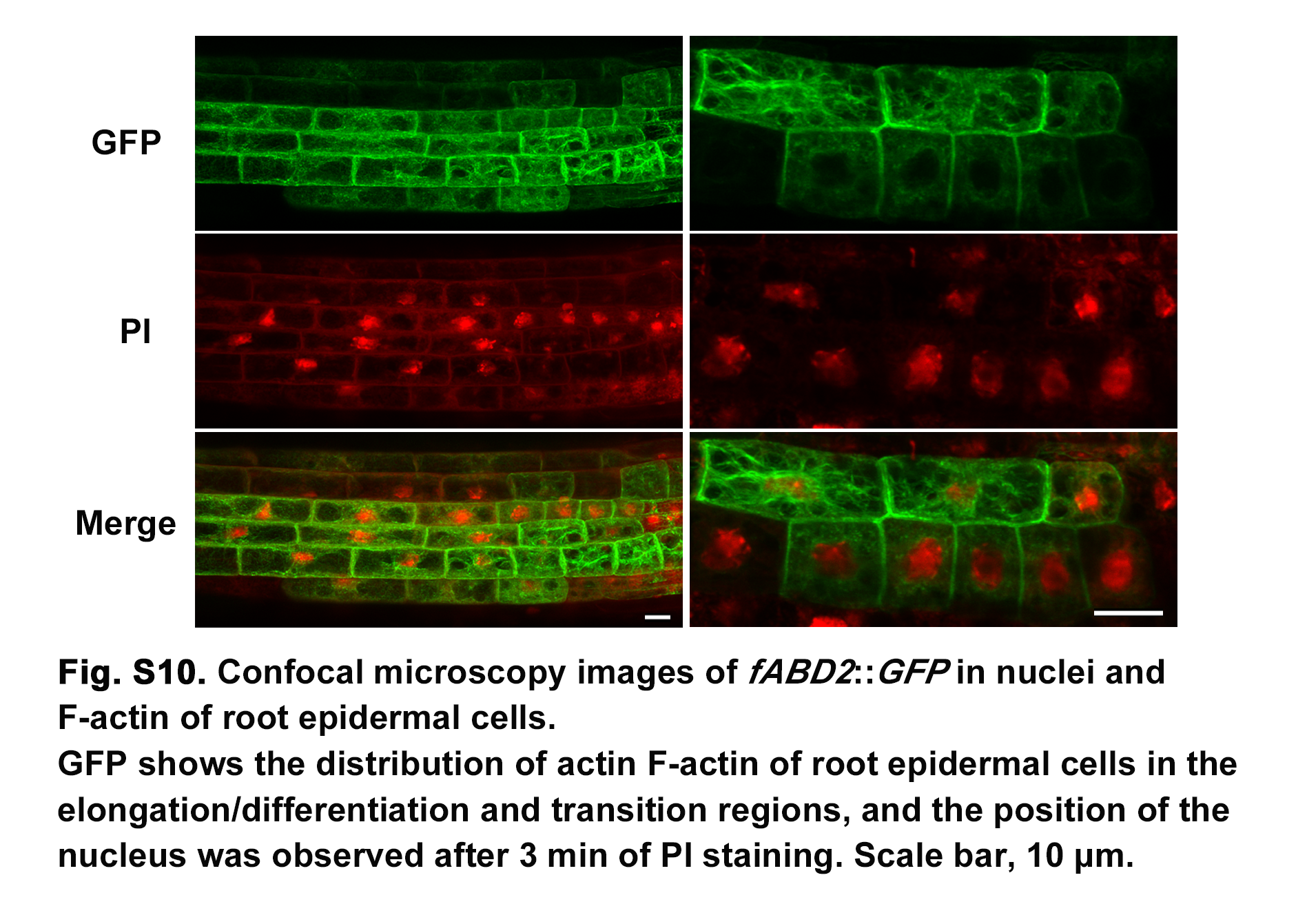

Supplement: S10 Fig — GFP shows the distribution of actin F-actin of root epidermal cells in the elongation/differentiation and transition regions, and the position of the nucleus was observed after 3 min of PI staining. Scale bar, 10 μm. (TIF) [file pgen.1010338.s010.tif]
