# Supplementary material for: Actin depolymerizing factor ADF7 inhibits actin bundling protein VILLIN1 to regulate root hair formation in response to osmotic stress in Arabidopsis
Source: PLoS Genet. 2022 Sep 12;18(9):e1010338. doi: 10.1371/journal.pgen.1010338 (PMC9499291; doi:10.1371/journal.pgen.1010338)
Supplement: S1 Table — (DOCX) [file pgen.1010338.s011.docx]

Table S1. Primers used in this study.

| *18S*-qPCR-F | CGGCTACCACATCCAAGGAA |
| --- | --- |
| *18S*-qPCR-R | GCTGGAATTACCGCGGCT |
| *ADF7*-qPCR-F | AGGATAGACGGGCAACAAGTGG |
| *ADF7*-qPCR-R | GAGGCGGTGAAATCATCGTAAGT |
| *VLN1*-qPCR-F | CAGAAGCCAGTGACGGATATAG |
| *VLN1*-qPCR-R | TCCACTTTGGAAGTGCATAGAA |
| *VLN3*-qPCR-F | CCAAAGGCAACACGCATAAC |
| *VLN3*-qPCR-R | CCTGATGGCCAAGAGTCAAA |
| *ADF7*-F1 | catcgttttggctcattttc |
| *ADF7*-R1 | TAGGAAAAGTTGCTAGAT |
| *ADF7*-F2 | acgatctatatctctatcatcgatc |
| *ADF7*-R2 | tccaagcttgggacgacagctgcta |
| *VLN1*-F1 | AAAAGTGGCTTGGAGATTTGG |
| *VLN*1-R1 | ATGGAGTCTGAGTTTGTTCTTGG |
| *VLN1*-F2 | AGGATGGAAAATTTTCAGGTG |
| *VLN1*-R2 | TTAGAAAAGATGAAGAGATATTTTGAG |
| *VLN1*-F3 | ATGTCTAGGCTAAGTAAAGACATTG |
| Promoter *VLN1*-F | GAACCATATGGAGATTAATGACG |
| Promoter *VLN1*-R | CTATACCCAAACAAAAATGGTTT |
| Promoter *ADF7*-F | CTGCAGTGCTCCACTCCACTG |
| Promoter *ADF7*-R | CCCCCGGGGAAGAAGATTTTGCTC |
